# Assessing signatures of selection through variation in linkage disequilibrium between taurine and indicine cattle

**DOI:** 10.1186/1297-9686-46-19

**Published:** 2014-03-04

**Authors:** Ana M Pérez O’Brien, Yuri T Utsunomiya, Gábor Mészáros, Derek M Bickhart, George E Liu, Curtis P Van Tassell, Tad S Sonstegard, Marcos VB Da Silva, José Fernando Garcia, Johann Sölkner

**Affiliations:** 1 University of Natural Resources and Life Sciences, Vienna, Austria; 2 São Paulo State University (UNESP), Araçatuba, São Paulo, Brazil; 3 The Roslin Institute, Edinburgh, UK; 4 USDA-ARS, Beltsville, MD, USA; 5 Embrapa Dairy Cattle, Juiz de Fora, Minas Gerais, Brazil

## Abstract

**Background:**

Signatures of selection are regions in the genome that have been preferentially increased in frequency and fixed in a population because of their functional importance in specific processes. These regions can be detected because of their lower genetic variability and specific regional linkage disequilibrium (LD) patterns.

**Methods:**

By comparing the differences in regional LD variation between dairy and beef cattle types, and between indicine and taurine subspecies, we aim at finding signatures of selection for production and adaptation in cattle breeds. The VarLD method was applied to compare the LD variation in the autosomal genome between breeds, including Angus and Brown Swiss, representing taurine breeds, and Nelore and Gir, representing indicine breeds. Genomic regions containing the top 0.01 and 0.1 percentile of signals were characterized using the UMD3.1 *Bos taurus* genome assembly to identify genes in those regions and compared with previously reported selection signatures and regions with copy number variation.

**Results:**

For all comparisons, the top 0.01 and 0.1 percentile included 26 and 165 signals and 17 and 125 genes, respectively, including *TECRL*, *BT.23182 or FPPS*, *CAST, MYOM1, UVRAG* and *DNAJA1*.

**Conclusions:**

The VarLD method is a powerful tool to identify differences in linkage disequilibrium between cattle populations and putative signatures of selection with potential adaptive and productive importance.

## Background

When a part of the genome that confers enhanced fitness or productive ability is preferentially kept in a population by increasing the frequency of favorable alleles, neutral loci that surround this region and that are in linkage disequilibrium (LD) with it, are also retained, thus driving the frequency of particular haplotypes in the region towards fixation in a pattern that decays progressively with distance from the causative location [1-4]. Such a selective sweep can be detected by reduced haplotype diversity and a different LD pattern when compared to those of the surrounding background [2,5]. Characterizing regions that are affected by selection may enable inferences on the functionality of a genomic region and possibly the effects of specific genes or gene combinations on specific traits [3-6].

Indicine (i.e., *Bos primigenius indicus*) cattle have been bred for adaptation to tropical and marginal production environments [7,8], while taurine (i.e., *Bos primigenius taurus*) cattle have been intensively selected for production in temperate regions of the world [5,7,9]. Analyzing differences between these two sub-species of cattle and comparing breeds selected for different purposes (milk or beef) within these subspecies may yield insights into genomic regions that are impacted by these differences in adaptation and productivity traits associated with these two groups of cattle [5]. The amount of LD that exists in genomic regions within a population is a key parameter to trace selective sweeps [2,3] and differences in decay of LD between bovine populations have been reported [9-12].

Analyses based on the study of regional variation of LD within a population compared to their background LD level, and the contrast of the regions with the same analyses in other populations, allow the assessment of signals of differential selection, also called signatures of selection (SS), in different cattle breeds. In addition, a high coincidence between SS and copy number variants (CNV) has been reported for the human Hapmap populations [13], which suggests that selection mechanisms may possibly act through copy number differences [14]. Indeed, a study of the effects of CNV on gene expression in Drosophila identified several potential outcomes of gene copy number variation, including the possibility that gene expression increases, decreases or remains stable as copy number fluctuates [15]. Thus, it is of interest to compare SS obtained via analysis of LD variation with reported CNV for the bovine genome [16,17].

## Methods

### Data

A total of 108 Nelore (NEL), 29 Gir (GIR), 33 Angus (ANG), and 85 Brown Swiss (BSW) individuals were genotyped using the Illumina BovineHD BeadChip (HD chip) [18]. The samples used were either derived from previous studies, approved by local ethical committees, or obtained from AI centers through their routine practice so no further ethical approval was required for the present analysis. Only autosomal SNPs were included in the analysis, resulting in approximately 735 000 SNPs. Quality control measures were calculated using the PLINK software [19,20] separately for each breed; parameters and thresholds used were a SNP minor allele frequency of at least 5%, a genotype call rate of at least 90%, both per SNP and per animal, and a Hardy-Weinberg equilibrium z-test with p > 10^-6^. In addition, the population was pruned for close relationships using the identity-by-state (IBS) relationship matrix, or in other words the pairwise genomic kinship coefficient as proposed by Leutenegger et al. [21], estimated with the GenABEL R package *ibs* function [22] and removing one of the individuals from a pair with an IBS > 0.8 (this limit was defined experimentally by assessing IBS relationships of 20 half-sibs). Final SNP counts and numbers of individuals used in the analyses are in Table 1.

**Table 1:** Number of individuals and SNPs per breed in the final data set

**Breed**	**Individuals**	**SNPs**
ANG	31	575082
BSW	79	550837
GIR	25	466953
NEL	100	448407

Individuals included and number of SNPs left for analysis in the final dataset after quality control was performed separately for each breed.

### Grouping

A total of six pair-wise comparisons between the four breeds were conducted. These comparisons included differences between the indicine and taurine (I/T) subspecies, differences between dairy and beef (D/B) breed types, and both subspecies and breed type differences (I/T, D/B). Specifically, the six comparisons were NEL/ANG (I/T), GIR/BSW (I/T), GIR/NEL (D/B), BSW/ANG (D/B), GIR/ANG (I/T, D/B), and BSW/NEL (I/T, D/B). Since the method applied here requires using common SNPs, i.e. SNPs that segregate in both breeds compared, for each comparison the coincident SNPs after quality control were extracted. The number of SNPs used in each analysis is in Table 2.

**Table 2:** Number of common SNPs in each breed comparison after quality control

**Comparison**	**SNPs**
NEL/ANG	338364
NEL/BRS	328305
GIR/NEL	384474
GIR/ANG	341857
GIR/BSW	331865
BSW/ANG	498788

### Principal component analysis

To have an overview of the population structure pertaining to the individuals and breeds included in the study, a Principal Component Analysis (PCA) was carried out using the IBS matrix generated with GenABEL [22], by converting the calculated genomic kinship coefficients to squared Euclidean distances that capture the differences between individuals via classical multidimensional scaling [23] in *n-1* dimensional spaces (where n represents the number of samples) of *n* eigenvectors, by applying the *cmdscale* function from the R ‘stats’ package v.3.0.1 [24].

### LD decay

To provide an insight about the overall levels of LD in the different breeds, genome-wide pairwise r^2^ values of SNPs separated by a maximum distance of 100 kilobases (kb) (average SNP distance was 7.9 kb), were calculated and graphed using R [24] and PLINK [19,20] software.

### VarLD

VarLD is a program for quantifying differences in genome-wide LD patterns between populations [13]. The software quantifies for each window of 50 SNPs the signed r^2^ of all pairwise comparisons and a square matrix is built with the results, representing a correlation matrix between all SNPs [25]. Equality between the elements of the two matrices is estimated by comparing the extent of departure between their respective ranked eigenvalues after eigen-decomposition of each matrix [25]. A raw VarLD score is assigned for the window as the trace of the difference between the respective diagonal matrices with the sorted eigenvalues in descending order [25]. The magnitude of this score gives a measure of the degree of dissimilarity between the correlation matrices and is used to quantify the extent of regional LD differences between the populations [25,26]. Positive selection for genes in a genomic region from a specific population is likely to produce a different LD pattern in that region when compared to a non-selected population, which leads to the identification of the region [2,3,6,26].

The methodology used to calculate VarLD scores is described in more detail in [13,25,26]. In short, permutation is used to obtain a Monte Carlo statistical significance and the scores are standardized (*S*_*i*_*’*) to center the distribution of the scores around a mean of zero and a standard deviation of one, helping to avoid bias in the raw VarLD scores due to differences in the size of the windows in terms of base-pairs (bp) and the populations being compared having different background LD levels. The software uses sliding windows and we applied windows containing 50 SNPs and a step-size of one SNP, following Teo et al. [13]. A window was flagged as a putative SS (SS region) if the associated score *S*_*i*_*’* was greater than or equal to the score at the 99.99^th^ percentile and 99.9^th^ percentile of all scores across the genome. The middle position of the first window in an identified SS was taken as the starting point of a signal, and the end position was the middle of the final window in the SS.

### Assigning signals to a breed

To assess which breed showed a selective sweep in a particular region with extreme *S*_*i*_*’*, we graphed LD heatmaps of the r^2^ between all SNPs from the identified SS region, using PLINK [19,20] and R [24]. Since the levels of r^2^ differed greatly between the two breeds on each comparison group it was relatively easy to determine the origin of a sweep by assigning it to the breed with the higher LD levels in the region.

To have an additional evaluation of the LD differences between the breeds included in the identified SS regions, we estimated the average r^2^ of SNPs in windows of 200 kb, with a step-size of 20 kb, discarding any windows that included less than 50 SNPs, and then graphed the results using R [24]. Only the graphs corresponding to the regions explored in detail in this publication are presented, together with graphical representation of the VarLD scores in these candidate SS regions.

### Gene identification

After the SS candidate regions were defined, we extracted details on these regions, including comparison group, chromosome, and bp position (middle position of the starting and ending windows included in the peak). Then, the SS regions were sorted by chromosome and bp position, and common signals across comparison groups were highlighted. To identify genes possibly associated with the SS regions, we compared the bp position of the regions to the position of the genes listed from the Ensembl Biomart Tool [27] for the UMD3.1 *Bos taurus* genome assembly [28,29], and extracted a list of genes having a common position with the SS regions.

### Confounding flagged regions with CNV regions and previously reported SS

Regions that were flagged by the above method were compared to the latest CNV reports by Bickhart et al. [16] and Hou et al. [17] on the bovine genome to detect common regions between VarLD SS and variations in copy number. The resulting signals were also compared to previously published SS using different methodologies and SNP densities. Information from the supplementary files of these publications was used for the comparisons.

## Results

**Figure 1:**
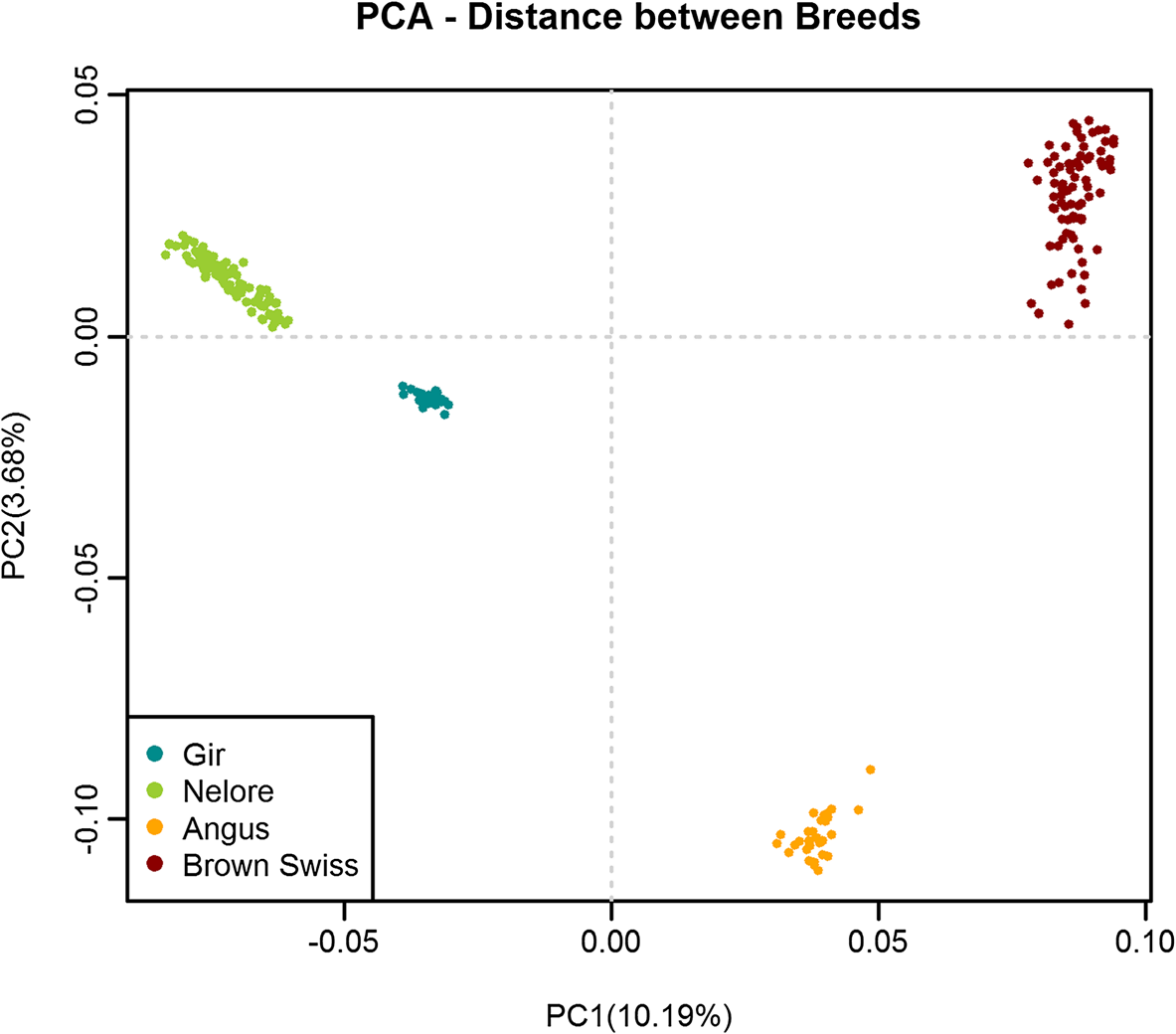
**Principal components diagram based on the genomic kinship coefficients between individuals.** Principal Component Analysis derived from the identity-by-state genomic relationship matrix between all individuals from the four studied breeds, showing the first two principal components (PC) and the variance explained by each component in parenthesis on the corresponding axis.

**Figure 2:**
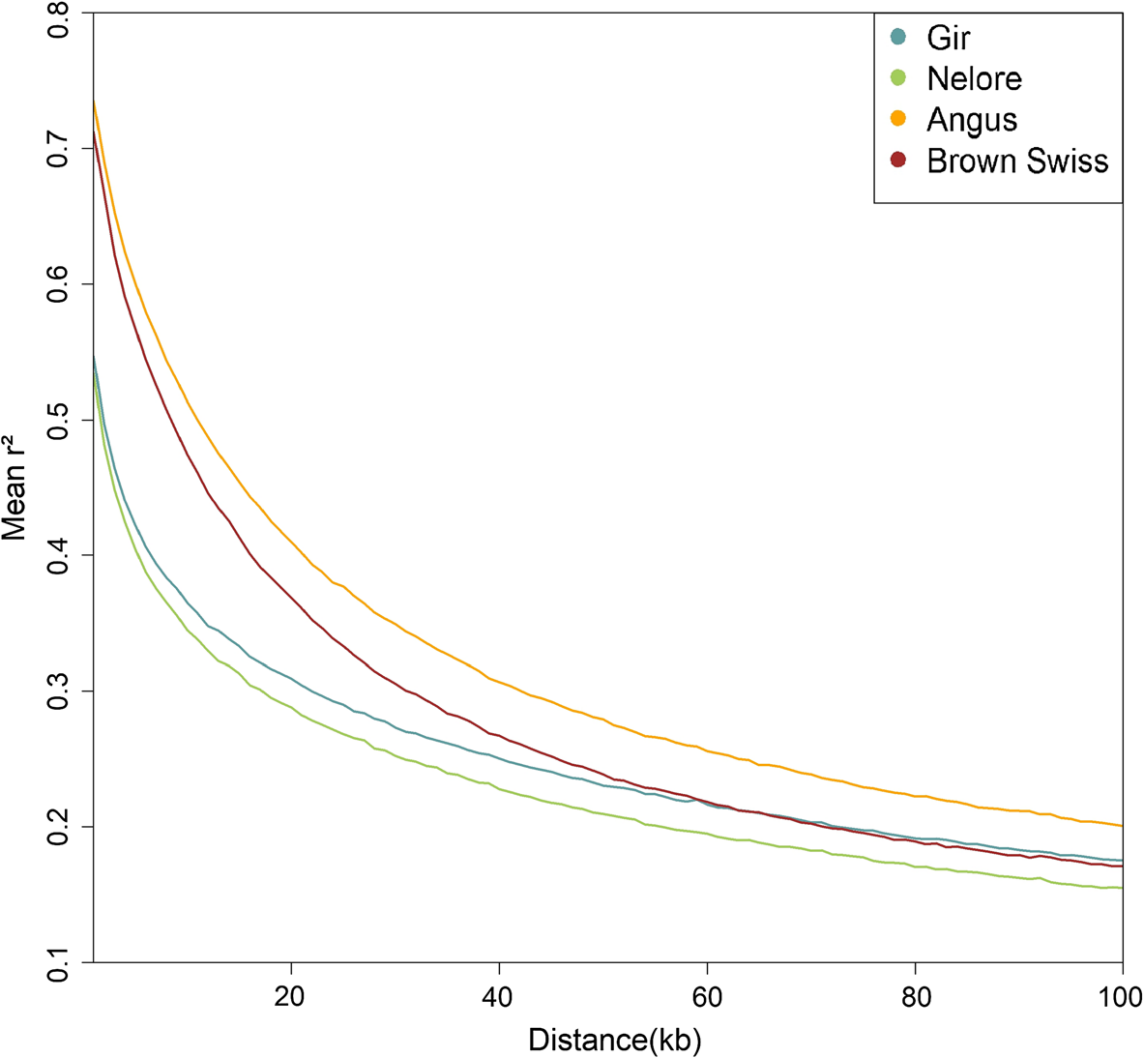
**LD decay up to 100 kb for the four studied breeds.** Average LD decay (r^2^) from 0 to 100 kb for each of the four breeds included in the analysis.

The PCA results (Figure 1) show that the first Principal Component (PC) explaining 10.2% of the SNP variation clearly separates the taurine and indicine populations, while the second PC explaining 3.7% of the variance divides each subspecies separating the breeds correctly. The patterns of dispersion also indicate that the two indicine breeds are genetically closer to each other and have lower within breed variance as compared to the taurine breeds. The results of LD decay up to a distance of 100 kb for the four breeds are in Figure 2. The pattern of decay shows higher LD at short distances for the taurine than the indicine breeds, particularly for Angus, reaching an average r^2^ of 0.3 at a distance of almost 40 kb, while both indicine breeds showed a faster decay, reaching an average r^2^ of 0.3 at approximately 20 kb.

**Figure 3:**
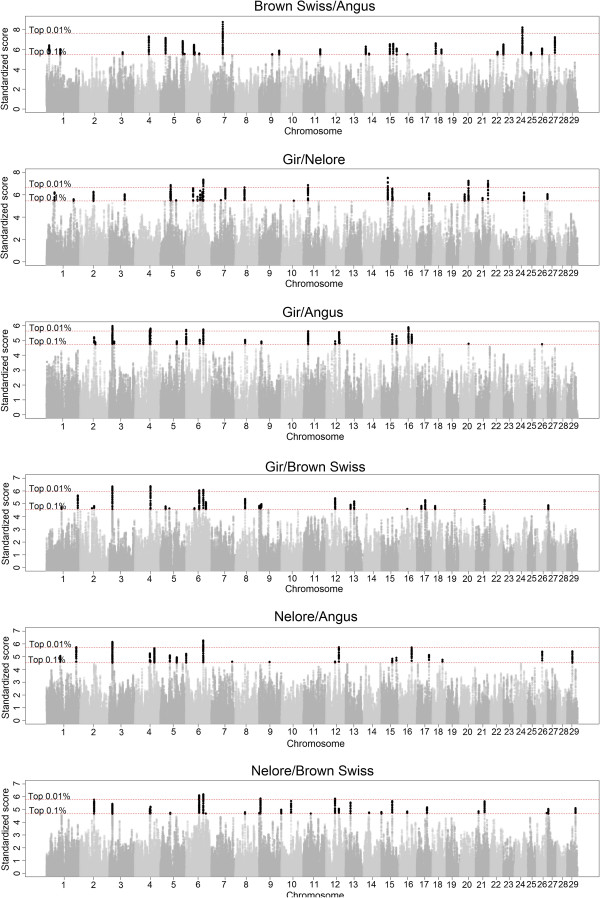
**Genome-wide VarLD analysis of the six breed comparisons.** Genome-wide plot of VarLD scores for all comparisons using the 99.99 and 99.9 percentiles of the standardized scores across the genome as thresholds.

The genome-wide distribution of standardized VarLD scores for the six comparison sets is in Figure 3. Strong SS were confined to narrow regions of the genome. The most distinct peaks were observed for the ANG/BSW and the GIR/NEL comparisons, which show that the largest VarLD scores are found when comparing different production types within a subspecies. This result is confirmed by the differences in the percentile distributions between the six comparison sets (Table 3), which shows higher 0.1 and 0.01 percentile scores for these two comparisons (ANG/BSW and GIR/NEL).

**Table 3:** Top percentile scores for each breed comparison

**Comparison**	**Top 0.01%**	**Top 0.1%**
NEL/ANG	5.73	4.53
NEL/BRS	5.77	4.66
GIR/NEL	6.68	5.46
GIR/ANG	5.62	4.73
GIR/BSW	5.94	4.56
BSW/ANG	7.63	5.48

Top 0.01 and 0.1 percentile limits of the standardized scores for each comparison.

For the top 0.01 percentile scores across all comparisons, 26 signals were found. Six SS were identified in more than one comparison and 17 genes were associated with these signals. For the top 0.1 percentile scores, 165 signals were detected, covering 10.76 Mb and representing 0.43% of the autosomal genome. Combining the SS shared across several comparison analyses, a total of 42 regions were identified with 125 genes related to these genomic positions (see Additional file 1).

**Figure 4:**
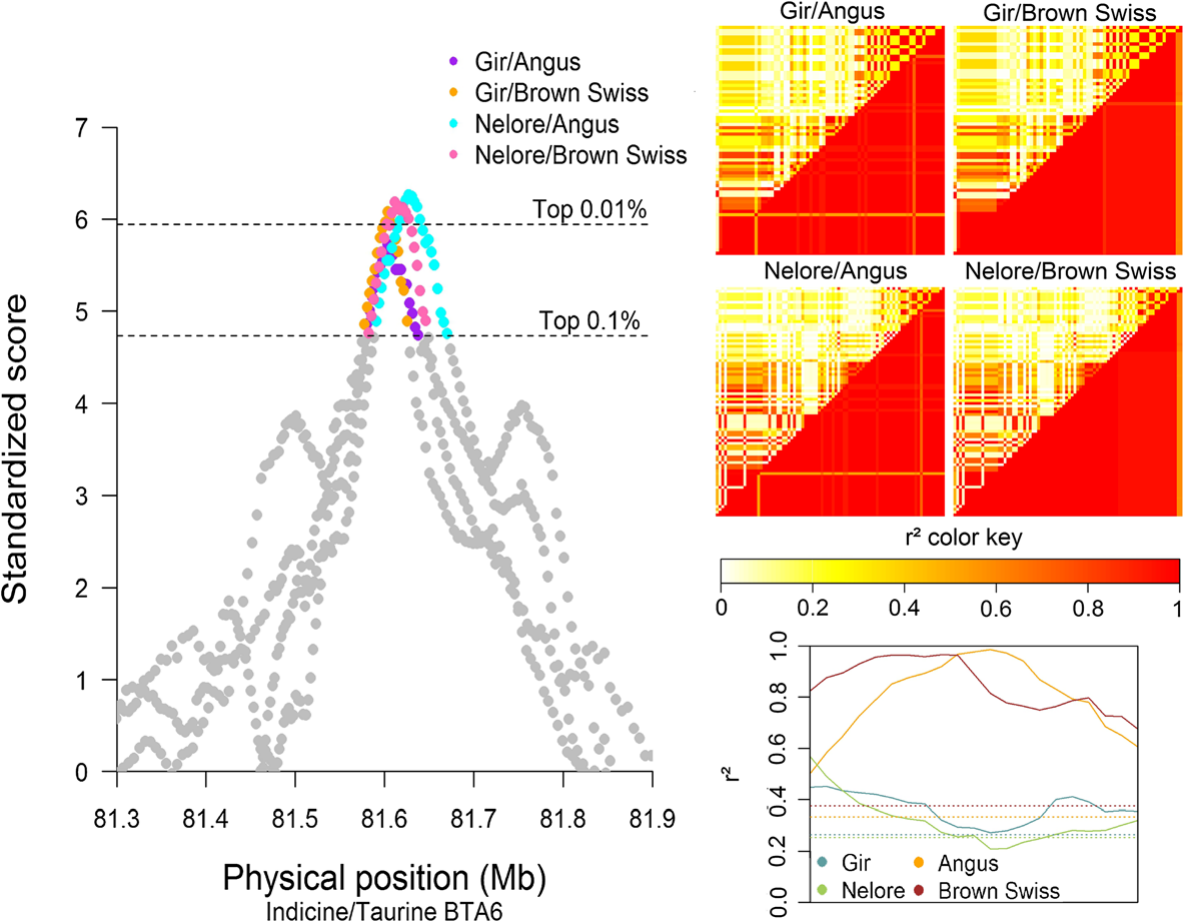
**Graphical results for the signal on BTA6 around 81.6 Mb, from the four indicine/taurine comparisons.** VarLD graph, LD heatmaps, and LD variation graphical results for the region showing a signal in all indicine/taurus comparisons on BTA6, between 81.5 and 81.7 Mb, inside the annotated boundaries of the *TECRL* (ENSBTAG00000024826) gene; the VarLD scores and LD variation analysis graphs represent the region between 81.4 to 81.8 Mb; the individual comparison heatmaps represent only the identified selection signature region, and on each heatmap the indicine breed is represented on the upper diagonal and the taurine breed on the lower diagonal.

For the I/T comparisons, detailed results for a signal that was found on BTA6 at 81.5-81.7 Mb and was shared across the NEL/ANG, NEL/BSW, GIR/ANG and GIR/BSW analyses are shown in Figure 4. This signal lies within the annotated boundaries of the *TECRL* (*trans-2,3-enoyl-CoA reductase-like*) gene (ENSBTAG00000024826) [29]. For this region, the two taurine breeds showed sustained high levels of LD, indicating a selective sweep in both these breeds. In addition, a loss of CNV, a type of variation caused by loss of genetic material due to deletions, was observed in this region, between 81.46 and 81.58 Mb, encompassing 71 SNPs and 120 kb.

**Figure 5:**
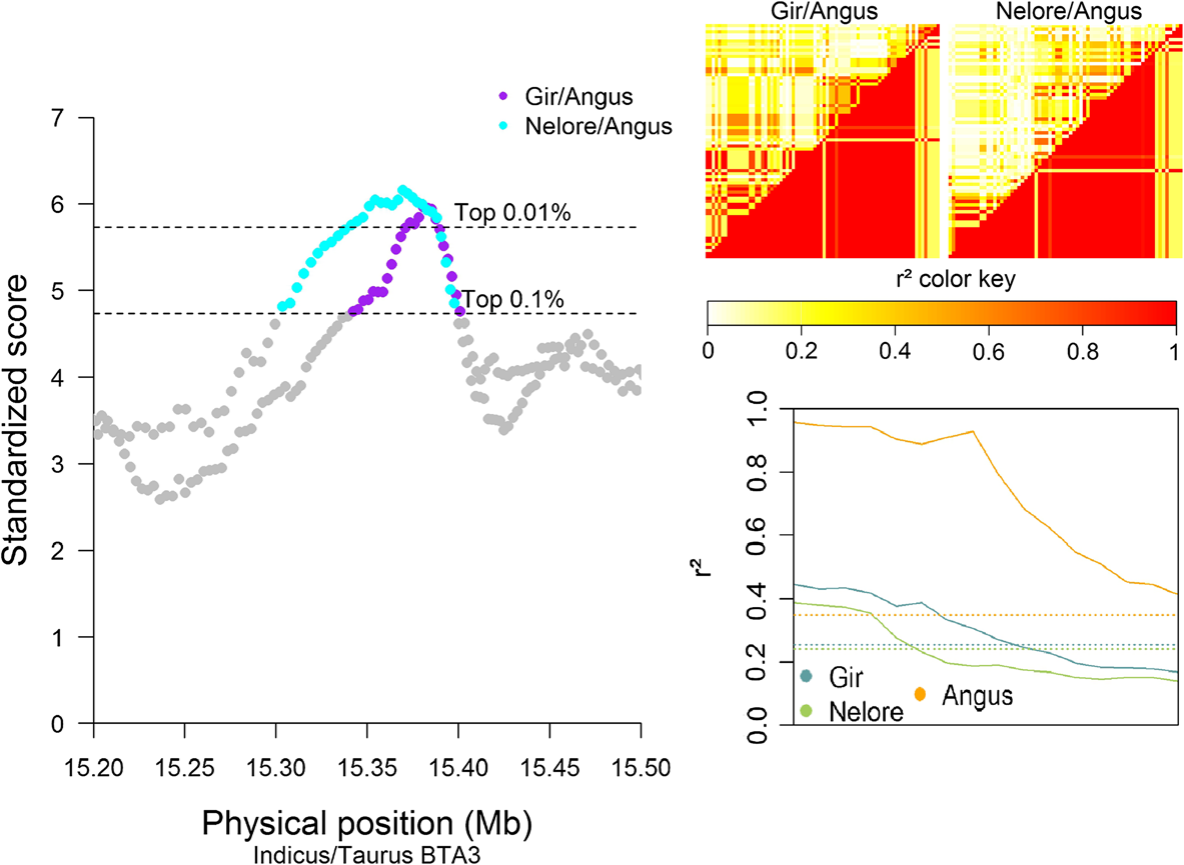
**Graphical results for the signal on BTA3 around 15.37 Mb, from two indicine/taurine comparisons.** VarLD graph, LD heatmaps, and LD variation graphical results for the region showing a signal in the Gir/Angus and Nelore/Angus comparison groups on BTA 3 between 15.34 and 15.39 Mb, overlapping with the annotated boundaries of the *BT.23182* gene (ENSBTAG00000003948); the VarLD scores and LD variation analysis graphs represent the region between 15.2 and 15.5 Mb; the individual comparison heatmaps represent only the identified selection signature region, with the Angus breed shown on the lower diagonal.

The NEL/ANG and GIR/ANG comparisons identified a SS on BTA3 between 14.9 and 15.5 Mb, with a peak between 15.37 and 15.39 Mb (Figure 5). When assessing the LD behavior of the three breeds involved in these comparisons, we found that the signal corresponded to a region with extended high LD in the ANG breed near the *FPPS_BOVIN* or *BT.23182* (ENSBTAG00000003948) [29] gene.

**Figure 6:**
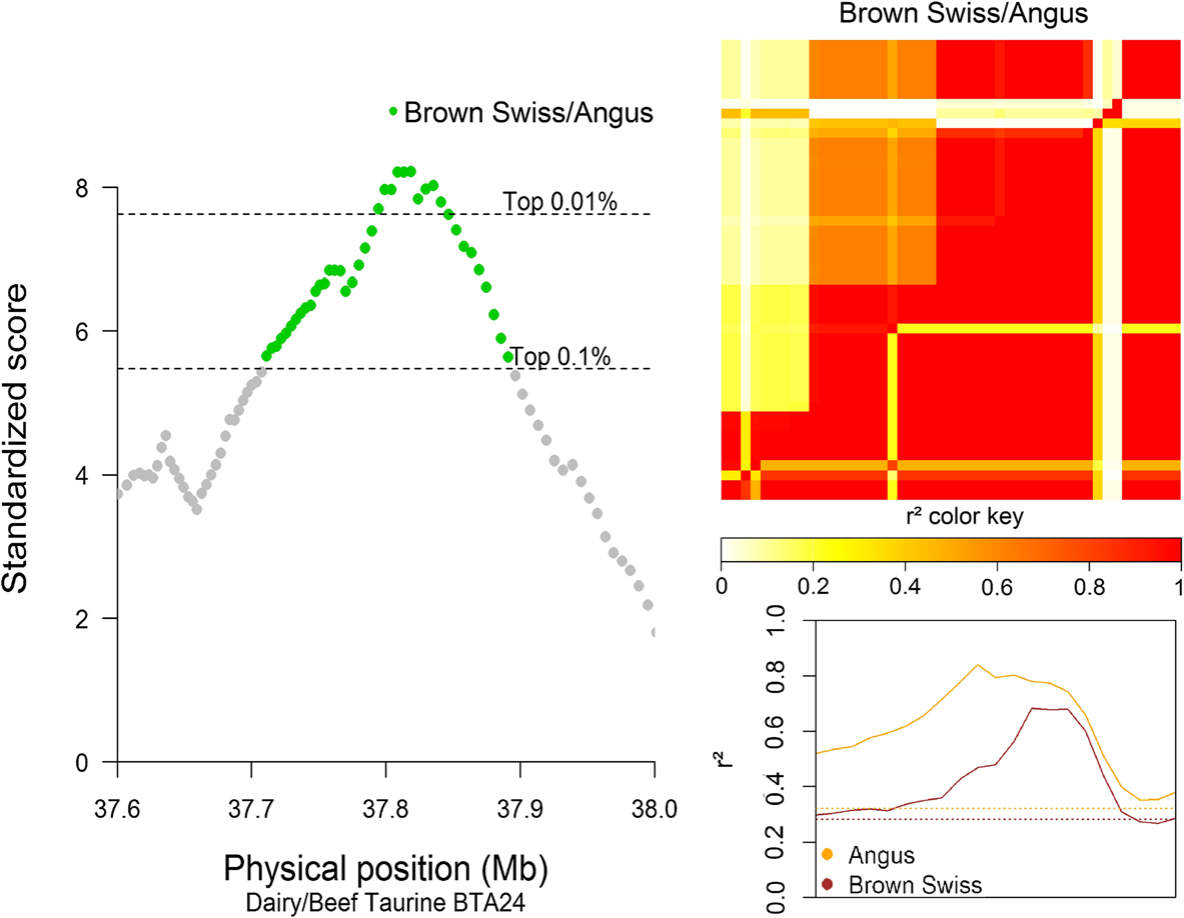
**Graphical results for the signal on BTA24 around 37.8 Mb, from the taurine dairy/beef comparison.** VarLD graph, LD heatmaps, and LD variation graphical results for the region showing a signal in the Brown Swiss/Angus comparison group on BTA24, between 37.79 and 37.84 Mb, inside the annotated boundaries of the *MYL9_BOVIN* (ENSBTAG00000016024) and the *MYL12B_BOVIN* (ENSBTAG00000026266) genes; the VarLD scores and LD variation analysis graphs represent the region between 37.6 and 38 Mb; the individual comparison heatmap represents only the identified selection signature region, with the Angus breed on the lower diagonal.

**Figure 7:**
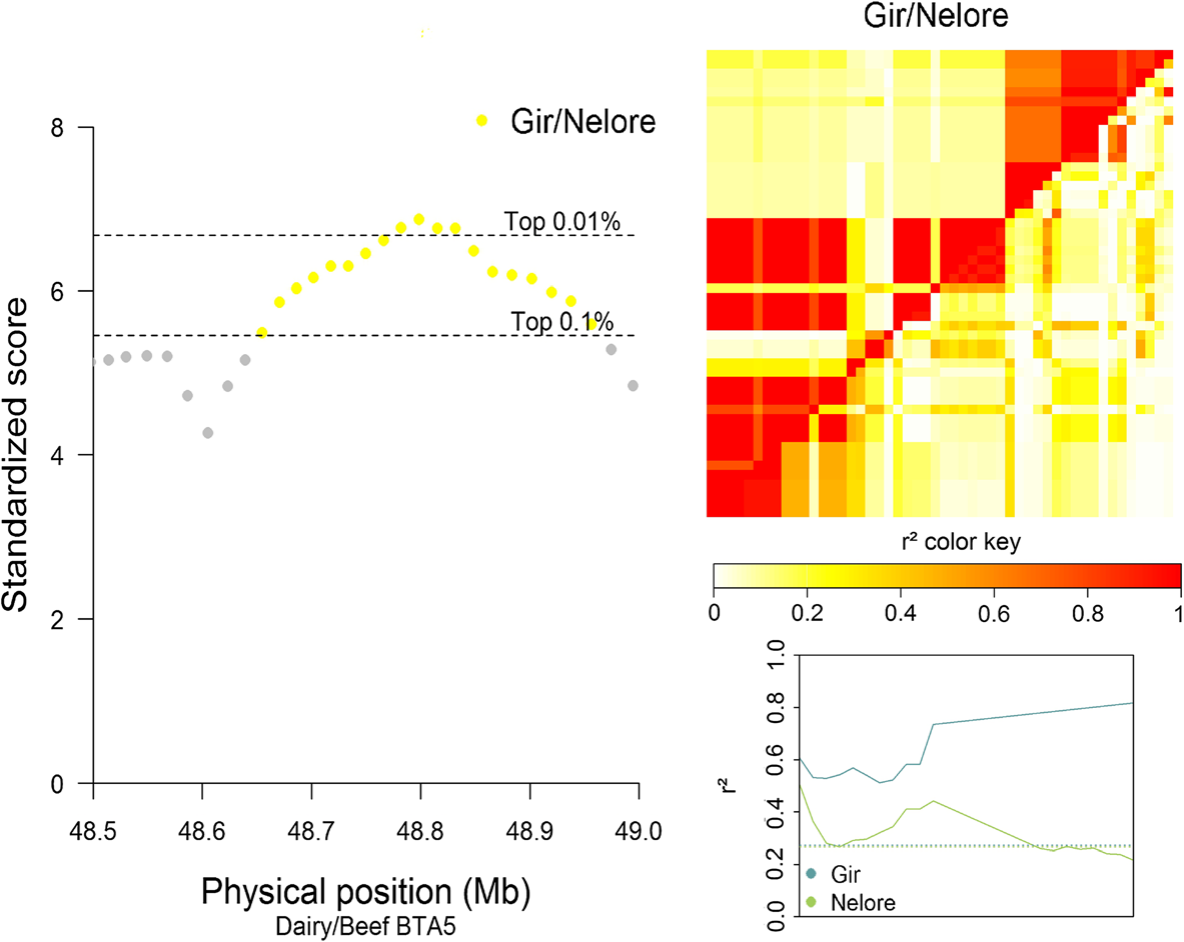
**Graphical results for the signal on BTA5 around 48.8 Mb, from the indicine dairy/beef comparison.** VarLD graph, LD heatmaps, and LD variation graphical results for the region showing a signal in the Gir/Nelore comparison group on BTA5, between 48.5 and 49.1 Mb, inside the annotated boundaries of the *LEMD3* (ENSBTAG00000039435) gene; the VarLD scores and LD variation analysis graphs represent the region between 48.5 and 49 Mb; the individual comparison heatmap, represents only the identified selection signature region, with the Gir breed on the upper diagonal.

For the D/B comparisons, the strongest signals were observed within subspecies, and primarily from taurine breed comparisons. For the taurine D/B comparison, one of the signals with the highest VarLD score was located on BTA24, between 37.79 and 37.84 Mb. This region (Figure 6) includes the annotated boundaries of the *MYL9* (*myosin, light chain 9, regulatory*) and the *MYL12B* (*myosin, light chain 12B, regulatory*) genes [29], and a high LD level between the SNPs in this region indicates that the SS is associated with the ANG breed. For the indicine D/B comparison, a signal on BTA5 between 48.5 and 49.1 Mb overlapped with the *LEMD3* (*LEM domain containing 3)*[29] gene (see Figure 7), and further analysis assigned this sweep to the Gir breed.

Thirty-four signature signals from the top 0.1 percentile were found in regions that contained reported bovine CNV [16,17], and genes located in these regions are presented in Table 4. These 34 regions cover 1.84 Mb or 0.07% of the autosomal genome, and many of the CNV positions coincided between the two reports [16] and [17] even though the authors have used different type of data as source of information for CNV discovery, sequence and SNPs, respectively. Information about several other candidate genes identified in this study through the VarLD methodology and that were previously identified in other cattle SS studies are presented in Additional file 2.

**Table 4:** Common signals between VarLD signals and bovine CNV

**Comparison**	**Chr**	**Signal position (bp)**	**CNV position (bp)**	**Author**	Genes (*)
GIR/BSW	1	70,765,503:70,792,506	70,749,256:70,782,268	[16,17]	LMLN
**NEL/BSW**	**3**	15,271,777:15,311,541	**15,239,708:15,333,979**	[16]	BT.58583
**NEL/ANG**		15,299,719:15,400,121		[16,17]	
**GIR/BSW**	4	73,396,066:73,553,054	**73,525,485:73,535,374**	[17]	ZNF804B
**GIR/ANG**		73,468,102:73,561,003			
**NEL/BSW**		73,483,619:73,529,284			
**NEL/ANG**		73,512,503:73,542,817		[16,17]	
GIR/NEL	5	48,655,139:49,351,919	49,247,629:49,260,811	[16,17]	TBC1D30
**GIR/ANG**	6	1,129,004:1,201,370	**1,193,082:1,294,965**	[16,17]	-
**NEL/ANG**		1,169,452:1,207,911			-
GIR/NEL	6	54,209,812:54,244,211	54,230,479:54,239,347	[16,17]	-
GIR/NEL	6	66,750,850:66,787,589	66,752,193:66,763,697	[16,17]	-
GIR/NEL	6	81,372,213:81,431,979	81,418,922:81,428,078	[16,17]	-
**GIR/BSW**		81,574,642:81,625,162	**81,459,939:81,580,677**		TECRL
**GIR/ANG**		81,580,533:81,637,706			
*BSW/ANG*	7	*53,792,514:53,993,49*	53,905,353:53,952,520	[16,17]	PCDHB4
			53,966,895:54,000,730	[17]	PCDHB6
					PCDHB7
GIR/NEL	7	65,840,794:65,909,733	65,783,735:65,871,908	[16,17]	-
**GIR/BSW**	8	46,292,757:46,328,656	**46,318,922:46,341,524**	[16,17]	C8H9orf135
**GIR/ANG**		46,309,661:46,327,604			
**NEL/BSW**		46,312,788:46,316,213			
NEL/ANG	9	49,905,617:49,915,316	49,985,406:50,007,670	[17]	ASCC3
**GIR/BSW**	12	41,634,760:41,915,116	**41,713,393:41,718,971**	[16,17]	-
**NEL/BSW**		41,664,252:41,803,562			-
**GIR/BSW**	12	41,634,760:41,915,116	**41,806,286:41,835,914**	[16,17]	-
**GIR/ANG**		41,788,070:41,814,404			
*GIR/ANG*	12	*41,987,029:42,007,452*	41,947,274:41,976,705	[17]	
			41,992,061:42,022,492	[16,17]	-
NEL/ANG	12	60,197,449:60,278,968	60,210,227:60,213,095	[16,17]	-
NEL/BSW	15	663,684:677,309	547,375:843,231	[16,17]	-
**NEL/BSW**	15	51,762,198:51,925,201	**51,800,593:51,828,442**		RRM1
**NEL/ANG**		51,800,901:51,937,349			
GIR/NEL	20	40,153,984:40,330,326	40,254,029:40,255,874	[16,17]	ADAMTS12
*GIR/NEL*	21	*60,490,921:60,601,371*	60,520,424:60,545,084	[16,17]	-
			60,560,357:60,579,349		
**BSW/ANG**	23	375,470:629,385	**436,008:1,208,595**	[16,17]	KHDRBS2
		756,762:853,782			

The breed comparison where the signal came from, the chromosome number, the VarLD signal and CNV positions in bp, the author reporting the CNV, and the genes with a short description, are all reported here; several VarLD signals that coincide with the same CNV are indicated in bold characters, while several CNV concurring with one signal are indicated in italic characters; *Gene ID source: ENSEMBL: http://www.ensembl.org/.

## Discussion

The VarLD method has the potential to capture recent strong selection because LD breaks down quickly over longer distances and, thus, high LD over an extended region is likely the result of recent selection. The human populations that have been analyzed [26,30,31] have very similar extents and patterns of LD and differ from each other only in limited regions. Cattle populations differ from human populations because they have experienced very strong recent selection caused by breed formation and use of advanced reproductive technologies. This makes the comparison of LD between cattle breeds worthwhile [9]. Differences such as those observed here between indicine and taurine breeds in the rate of decay of LD with increasing distance have been previously reported but with lower marker densities [9,10,32,33]. Our analysis clearly shows that the pattern of LD decay is faster in the indicine breeds compared to the taurine breeds. This supports the use of higher SNP densities in the indicine breeds, both for LD analysis and differences in LD patterns, in order to capture the nature of genomic events that affect narrow regions by having SNPs sufficiently close to the cause of an event to show significant LD. In this study, the regions with the highest VarLD scores that we identified were very narrow, with the largest signal covering 696.78 kb and the smallest signal involving single SNPs, which confirms the benefit of using a high-density SNP beadchip for this approach.

The effect of ascertainment bias in the choice of SNPs for different SNP chip platforms has been discussed in the literature [34], but the HD chip was constructed using a larger number of indicine breeds and individuals in the reference population, and in general seems to perform better on *Bos indicus* individuals, than the Illumina BovineSNP50 BeadChip [18]. When the analysis was replicated using the 50Kchip SNPs, nine signals were found for the 0.1 percentile, covering 24.9 Mb of the autosomal genome, and ranging in size from 212.3 kb to 10.2 Mb (results not shown), with only four regions found in common with the analyses performed using the HD chip. This demonstrates a capacity for higher resolution analyses when using the HD chip.

### Highest scoring SS for different comparisons

In the I/T comparisons, the strongest signal identified in all breed contrasts was created by unusually high LD in the taurine breeds. The *TECRL*[29] gene encodes an enzyme that has an oxidoreductase activity on the CH-CH group of donors and other acceptors, and is directly involved in chemical reactions and pathways involving lipids [35]. The SS region containing *TECRL* also overlaps with a region in which a particular type of CNV with loss of nucleotides is commonly observed, which suggests a possible role of copy number differences being causative in selection processes. Because the selection signature was found in the taurine breeds and is directly related to lipid production in the body, this is a suggestive signature of artificial selection for production purposes.

The *FPPS_BOVIN*[29] gene, detected in a signal between ANG and both indicine breeds, is a gene involved in cholesterol (sterols) and steroid biosynthesis [35]. Lipid synthesis is a very important physiological function for both milk [36] and beef [37,38] production, and both taurine breeds have been selected intensively for these characteristics during the past decades [5,7,9].

The *LEMD3* gene [29], detected as a selective sweep in the Gir breed, is a specific repressor of the transforming growth factor beta (TGF-beta) receptor, activin, and BMP signaling, and is involved in negative regulation of skeletal muscle cell differentiation, which might have been selected for in Gir, a breed developed for milk production [35]. In humans, mutations leading to loss of function of this gene are associated with diseases causing sclerosing bone lesions and increased bone density, such as osteopoikilosis [39,40]. This selection signature was reported by Ramey et al. [41], between 48.67 and 48.9 Mb on BTA5, using an approach based on sliding windows estimations of minor allele frequency (MAF).

In the taurine D/B comparison, two genes possibly related with variation in muscle accretion were identified i.e. *MYL12A* and *MYL12B*[29]. *MYL12A* encodes a non-sarcomeric myosin complex component with calcium ion binding regulatory functions that are involved in signal transduction mechanisms, cytoskeleton formation, cell division and chromosome partitioning [35]. *MYL12B*[29] encodes a component of the Z-disc and the myosin II complex. Phosphorylation of MYL12B regulates the activity of non-muscle myosin II, resulting in higher MgATPase activity and the assembly of myosin II filaments. It is also involved in axon guidance processes, muscle contraction and regulation of muscle cell shape [35]. When extending detection of signatures of selection to the 0.1 percentile of VarLD scores, a third gene in this region overlapped with the signal: *MYOM1* (*myomesin 1*) [29], which encodes a 85 kDa protein that is a structural constituent of muscle. Together with its associated proteins, the MYOM1 protein interconnects the major structure of sarcomeres, the M bands and the Z discs, and is involved in muscle contraction [35]. *MYOM1* is one of the top 10 genes with preferential expression in muscle tissue [42] and has been associated with intramuscular fat content [43]. In addition, the most significant physiological and system development functions associated with genes involved in meat tenderness include skeletal and muscular system development and tissue morphology, both of which have been related with muscle contraction in the pig [43].

The *CAST* (*calpastatin isoform I*) gene (ENSBTAG00000000874) [29] identified on BTA7 between 98.44 and 98.58 Mb in the NEL/ANG comparison, with the signal originating from ANG, has been intensively studied in different breeds and selected for to improve meat tenderness and other traits associated with beef quality [44-50]. The gene encodes an endogenous calpain (calcium-dependent cysteine protease) inhibitor that is involved in the proteolysis of amyloid precursor proteins. The calpain/calpastatin system is involved in numerous membrane fusion events, such as neural vesicle exocytosis and platelet and red-cell aggregation, and it is hypothesized that it affects the expression of genes encoding structural or regulatory proteins [35]. Due to its capacity to prevent proteolysis [35], some polymorphisms in this gene have been shown to be associated with increased meat tenderness in beef cattle breeds [49].

The *protocadherin beta* gene cluster (*PCDHB4*, *PCDHB6*, *PCDHB7*, *PCDHB13*, among others) [29] was identified as having a selection signature in the taurine D/B comparison. This cluster encodes neural cadherin-like cell adhesion proteins that are integral plasma membrane proteins and most likely play critical roles in the establishment and function of specific cell-cell neural connections [35]. In addition, these proteins are involved in nervous system development, synapse assembly, and synaptic transmission [35]. As reported by MacGregor [51], *protocadherin II* contains a high-affinity cell surface binding site for Prion proteins and a number of *protocadherin* genes also function as tumor suppressor genes [52,53]. Three *protocadherin* genes, *protocadherin-psi1*, *PCDHB4* and *PCDHB6* were previously reported as a selection signature using an Fst approach [54] and were found to overlap with CNV regions.

The *UVRAG* (*UV radiation resistance associated*) (ENSBTAG00000016355) [29] gene located on BTA15 between 56.2 and 56.3 Mb, was found to have a selection signature in the comparison between the BSW and ANG breeds. This gene is associated with DNA repair and positive regulation of autophagy [35]. The human homologue of this gene [55] has been shown to complement the ultraviolet sensitivity of *xeroderma pigmentosum* group C cells [56] and encodes a protein with a C2 domain [57]. This protein activates a Beclin1 complex that promotes autophagy and suppresses the proliferation and tumorigenicity of human colon cancer cells [58].

The *DNAJA1* (*DnaJ (Hsp40) homolog, subfamily B, member 1*) (ENSBTAG00000045858) gene [29], located on BTA7 between 53.8 and 54 Mb, was identified in the comparison between the BSW and ANG breeds. It encodes a heat shock protein binding gene [35], which is a co-chaperone of the 70 kDa heat shock protein (Hsp70), and the *DNAJA1*/*Hsp70* complex directly inhibits apoptosis [59]. Because of its anti-apoptotic role, it has been considered as having an important role in meat tenderness in beef cattle. Association studies showed that this gene explained up to 63% of the phenotypic variability of tenderness in Charolais [60]. The selection signature identified in the *DNAJA1* gene could be a good indicator of selection for meat tenderness in the ANG breed during the last decades.

### Comparison of VarLD with other methods to detect selection signatures

Several genes previously reported using other methods to detect SS were also identified in our study. The first example is the *MSRB3 (methionine sulfoxide reductase B3*) gene (ENSBTAG00000044017) [29] located on BTA5 between 48.56 and 48.74 Mb for which a SS was found in the NEL/GIR comparison between 48.65 and 49.35 Mb, which was also found by Ramey et al. in Brahman populations [41]. This gene has been associated with the ‘long ear’ phenotype, which characterizes the Gir breed, and against which the Nelore breed has been strongly selected; this reveals a clear sign of differential selection between the indicine breeds [41]. *MSRB3* was first identified through a genome-wide association study as a candidate for a QTL involved in ear floppiness and morphology in dogs [61]; it is an indicator of strong artificial selection for a specific phenotype, and of the time at which the breed was formed.

A second example is the *PCSK4* (*proprotein convertase subtilisin/kexin type 4*) gene (ENSBTAG00000002305) [29] on BTA7. The SS was identified in the NEL/GIR comparison at 45.5 Mb. This gene was also reported in SS studies by [41] and [9] in Jersey and Santa Gertrudis breeds. It is responsible for serine-type endopeptidase activity, which is involved in acrosome reaction, binding of sperm to the zona pellucida, sperm capacitation, and fertilization, which are all key functions of male fertility [35].

A third example is the *TOX* (*thymocyte selection-associated high mobility group box*) gene located on BTA14 between 26.6 and 26.9 Mb (ENSBTAG00000004954) [29]. This gene encodes a possible bovine blood group antigen transcript. Blood group antigens have been shown to be under balancing selection in humans [62], and this gene was also reported to be under positive selection in the Normande and Montbéliarde French dairy breeds by Flori et al. [63].

Surprisingly, although haplotype-based and LD methods are expected to perform similarly [64], when the Rsb method for detecting selection signatures [65], which evaluates differences between breeds by estimating extended haplotype homozygosity (EHH) for each SNP location, was applied in our data, the results were quite different, and only two regions shared a signal. Another method, ΔDAF [66], which is based on the difference in the derived allele frequency between populations, was also tested and no common signals were identified. Considering the differences that the adopted LD method could have with other methods to identify SS, LD methods may detect regions that haplotype based-methods such as EHH [67], iHs [67], Voight’s iHs [68], and Rsb [65] might not detect, because genomic processes such as insertion/deletion (in/del) and other CNV produce LD patterns that may not be accounted for in the haplotype construction.

The fact that LD methods cannot deal with monomorphic SNPs, makes VarLD less sensitive for regions with completely fixed SNPs or with many fixed SNPs for one population. Such signatures might be detected using methods like smoothed Fst [9,69] and MAF-based [41] approaches.

### Comparison of VarLD results with CNV regions

Several of the identified selection signatures, especially for the indicine breeds, pointed to non-genic regions, including some CNV regions, such as (i) the signal on BTA6 between 66.75 and 66.78 Mb observed in the NEL/GIR comparison that coincides with a CNV on BTA6 between 66.75 and 66.76 Mb, and (ii) a CNV on BTA8 between 46.31 and 46.34 Mb that coincides with two overlapping SS observed in the GIR/BSW and GIR/ANG comparisons. These results suggest that different types of genomic variation, other than SNPs, may have a role in selection mechanisms. Given that CNV have been shown to influence gene expression through dosage-dependent interactions [15], it is possible that the identified VarLD regions correspond to selection for a specific gene copy number or for a certain duplicated paralog that is present in the duplication.

Across the whole genome, most CNV have evolved under neutral evolutionary pressures and their frequency and sequence context have been shaped by demographic events, mutation, and genetic drift [14,15,17]. However, CNV that are located in functional regions of overlapping genes, are mostly under purifying (negative) selection and only a few examples of positive selection on these CNV are known [15]. Regions that differ in copy number between subspecies can be informative about ancient adaptations that may have led to species-specific phenotypes. Recent copy number changes can be an indicator that artificial selection may have led to genetic and phenotypic differences between breeds.

In previous studies using the VarLD method to analyze human data, a large fraction of the top signals corresponded to CNV for some of the populations compared [13]. Comparing our signals from the top 0.1% VarLD scores to recently published reports on the detection of bovine CNV [16,17], we found that 20.6% of our signals overlapped with reported CNV. Since these signals cover only 0.43% of the genome and the CNV discovery sets included 2.1 and 5.6% of the genome, respectively [16,17], it is hypothesized that CNV are associated with differences in LD between populations and with selection processes [13-15].

## Conclusions

VarLD is a powerful tool to identify differences in LD between cattle populations and possible signals of directional selection between them. The strongest signals differentiate LD patterns between breeds within subspecies and seem to point towards very recent selection. The narrow signatures of selection peaks that were detected in this analysis seem to indicate that both the methodology and the SNP density applied were appropriate to identify genes that underlie the identified selective sweeps.

Some of the genes found in the I/T comparisons indicate potential adaptive signatures, while the D/B comparisons point out genomic regions related to production of milk and beef. A high number of the genomic regions identified with the VarLD method were shown to be associated with physiological pathways of adaptation and production processes, and some of the genes present in these regions have also been reported to coincide with signatures of selection in other species.

The fact that 20.6% of the top VarLD signals overlap with recently reported CNV regions, which cover less than 7.7% of the genome, is a strong indicator of the role of CNV in selection within a breed type. In contrast, it is surprising that results from previous studies using the same breed comparisons and partially overlapping data sets, which applied haplotype-based methods to detect signatures of selection, had almost no overlap with the signals we detected using the VarLD method.

## Competing interests

The authors declare that they have no competing interests.

## Authors’ contributions

JS and AMPO conceived and designed the study. AMPO performed data preparation, statistical analysis, and drafted the manuscript. YTU, GM and DMB performed data preparation and participated in the statistical analysis. GEL, CPV, TS, MVBDS, JFG, and JS helped in data acquisition, interpretation of results, and critical revision. FG and JS coordinated the collaborative efforts. All authors read and approved the final manuscript.

## Supplementary Material

Additional file 1 **Signals found for the top 0.01 and 0.1 percentile of VarLD scores.** Table with the signals found on the top 0.1 percentile of the distribution of VarLD scores, organized by chromosome and bp position along the genome; the breed comparison where the signal came from, the chromosome number, the starting and ending bp position and information for the genes spanning the regions under the signals are given in the table; additionally, signals that contain the 0.01 percentile are highlighted in yellow, and in regions that cover several genes, the genes underlying the highest scoring window are highlighted in blue. Click here for file

Additional file 2 **Common signals with other Signatures of Selection studies.** Common signals found between our analysis and previous signatures of selection regions reported in the literature; the breed comparison where the signal came from, the chromosome number, the base pair spanning position, the position of the common region, the reference to the authors and the gene name are given in the Table; several VarLD signals that coincide with the same signal from other studies are highlighted in yellow, while several signals from other authors that concur with one VarLD Signal are highlighted in blue [5,7,9,41,54,63,64,69-73]. Click here for file
