# Supplementary material for: Assessing signatures of selection through variation in linkage disequilibrium between taurine and indicine cattle
Source: Genet Sel Evol. 2014 Mar 4;46(1):19. doi: 10.1186/1297-9686-46-19 (PMC4014805; doi:10.1186/1297-9686-46-19)
Supplement: Additional file 1 — Signals found for the top 0.01 and 0.1 percentile of VarLD scores. Table with the signals found on the top 0.1 percentile of the distribution of VarLD scores, organized by chromosome and bp position along the genome; the breed comparison where the signal came from, the chromosome number, the starting and ending bp position and information for the genes spanning the regions under the signals are given in the table; additionally, signals that contain the 0.01 percentile are highlighted in yellow, and in regions that cover several genes, the genes underlying the highest scoring window are highlighted in blue. [file 1297-9686-46-19-S1.doc]

| **Comparison** | **Chr** | **bp-start** | **bp-end** | **Ensembl Gene ID** | ***Gene Name**** | **Gene Description**** | **Gene Location*** |
| --- | --- | --- | --- | --- | --- | --- | --- |
| BSW/ANG | 1 | 12,175,001 | 12,258,890 | ENSBTAG00000048165 | Known miRNA | Novel miRNA | 12,234,087-12,234,183 |
| GIR/NEL | 1 | 37,843,924 | 37,893,835 | ENSBTAG00000023652 | *PROS1* | Vitamin K-dependent protein S | 37,803,108-37,866,950 |
|  |  |  |  | ENSBTAG00000005155 | *BT.90528* | ARL13B | 37,875,395-37,952,674 |
| NEL/ANG | 1 | 64,425,687 | 64,443,609 | ENSBTAG00000045704 | *BT.90639* | Immunoglobulin superfamily member 11 precursor (IGSF11) | 64,357,122-64,488,105 |
| BSW/ANG | 1 | 64,426,469 | 64,435,916 |  |  |  | 64,357,122-64,488,105 |
| GIR/BSW | 1 | 70,765,503 | 70,792,506 | ENSBTAG00000044172 | *LMLN* | Leishmanolysin-like (metallopeptidase M8 family) | 70,742,636-70,784,188 |
|  |  |  |  | ENSBTAG00000014208 | *RPL35A* | 60S ribosomal protein L35a | 70,788,697-70,792,140 |
|  |  |  |  | ENSBTAG00000018025 | *BT.56204* | IQ domain-containing protein G | 70,792,452-70,838,105 |
|  |  |  |  | ENSBTAG00000045455 | *U6* | U6 spliceosomal RNA | 70,786,714-70,786,824 |
| GIR/NEL | 1 | 127,612,598 | 127,664,230 | ENSBTAG00000011524 | *XRN1* | 5'-3' exoribonuclease 1 | 127,591,937-127,699,862 |
| NEL/ANG | 1 | 139,616,236 | 139,744,615 | ENSBTAG00000010448 | *CPNE4* | Copine IV | 139,678,041-139,842,029 |
| GIR/BSW | 1 | 146,982,369 | 147,014,882 | ENSBTAG00000023907 | *BT.11942* | Collagen alpha-1(XVIII) chain | 146,989,244-147,040,968 |
| GIR/BSW | 2 | 55,263,565 | 55,266,085 | None | *-* | - | - |
| GIR/NEL | 2 | 62,798,921 | 62,930,142 | ENSBTAG00000018753 | *TMEM163* | Transmembrane protein 163 | 62,843,283-63,111,812 |
| GIR/ANG | 2 | 65,465,244 | 65,479,573 | None | *-* | - | - |
| NEL/BSW |  | 65,540,772 | 65,604,699 |  |  |  |  |
| GIR/BSW |  | 65,580,520 | 65,599,835 |  |  |  |  |
| GIR/ANG | 2 | 71,533,731 | 71,553,782 | ENSBTAG00000010599 | *C2H2orf76* | UPF0538 protein C2orf76 homolog | 71,473,546-71,561,045 |
| GIR/BSW | 3 | 14,993,410 | 15,069,437 | ENSBTAG00000020356 | *BT.45280* | Gon-4-like | 15,009,727-15,093,527 |
| NEL/BSW | 3 | 15,016,153 | 15,137,198 |  |  |  |  |
|  |  |  |  | ENSBTAG00000026613 | *MSTO1* | Protein misato homolog 1 | 15,093,505-15,097,679 |
|  |  |  |  | ENSBTAG00000001199 | *DAP3* | 28S ribosomal protein S29, mitochondrial | 15,106,137-15,145,083 |
| GIR/ANG | 3 | 15,016,365 | 15,067,190 | ENSBTAG00000020356 | *BT.45280* | gon-4-like | 15,009,727-15,093,527 |
|  |  |  |  | ENSBTAG00000026613 | *MSTO1* | Protein misato homolog 1 | 15,093,505-15,097,679 |
|  |  |  |  | ENSBTAG00000001199 | *DAP3* | 28S ribosomal protein S29, mitochondrial | 15,106,137-15,145,083 |
| NEL/ANG | 3 | 15,072,372 | 15,143,801 | ENSBTAG00000020356 | *BT.45280* | Gon-4-like | 15,009,727-15,093,527 |
|  |  |  |  | ENSBTAG00000026613 | *MSTO1* | Protein misato homolog 1 | 15,093,505-15,097,679 |
|  |  |  |  | ENSBTAG00000001199 | *DAP3* | 28S ribosomal protein S29, mitochondrial | 15,106,137-15,145,083 |
| NEL/BSW | 3 | 15,271,777 | 15,311,541 | ENSBTAG00000003954 | *BT.58583* | probable histone-lysine N-methyltransferase ASH1L | 15,176,831-15,353,404 |
| NEL/ANG | 3 | 15,299,719 | 15,400,121 |  |  |  |  |
|  |  |  |  | ENSBTAG00000003948 | *BT.23182* | Farnesyl pyrophosphate synthase ( FPPS) | 15,356,047-15,374,937 |
|  |  |  |  | ENSBTAG00000017056 | *PKLR* | Pyruvate kinase isozymes R/L | 15,399,755-15,408,994 |
| GIR/ANG | 3 | 15,342,320 | 15,400,959 | ENSBTAG00000003954 | *BT.58583* | probable histone-lysine N-methyltransferase ASH1L | 15,176,831-15,353,404 |
|  |  |  |  | ENSBTAG00000003948 | *BT.23182* | Farnesyl pyrophosphate synthase( FPPS) | 15,356,047-15,374,937 |
|  |  |  |  | ENSBTAG00000017056 | *PKLR* | Pyruvate kinase isozymes R/L | 15,399,755-15,408,994 |
| NEL/ANG | 3 | 15,523,608 | 15,552,545 | ENSBTAG00000020244 | *EFNA1* | Ephrin-A1 Ephrin-A1, secreted form | 15,521,494-15,528,093 |
| GIR/ANG | 3 | 23,662,912 | 23,670,826 | ENSBTAG00000003898 | *HMGCS2* | Hydroxymethylglutaryl-CoA synthase, mitochondrial | 23,643,772-23,667,741 |
| BSW/ANG | 3 | 64,166,604 | 64,177,456 | None | *-* | - | - |
| GIR/NEL | 3 | 73,241,643 | 73,288,967 | ENSBTAG00000005099 |  | Novel pseudogene | 73,263,144-73,263,914 |
| BSW/ANG | 4 | 66,034,317 | 66,065,574 | ENSBTAG00000014381 | *BT.25906* | Corticotropin-releasing factor receptor 2 | 66,062,161-66,090,181 |
| GIR/ANG | 4 | 70,487,854 | 70,570,667 | ENSBTAG00000043517 | *U6* | U6 spliceosomal RNA | 70,560,498-70,560,604 |
| GIR/ANG | 4 | 70,768,097 | 70,933,946 | None | *-* | - | - |
| NEL/ANG | 4 | 70,786,800 | 70,897,862 |  |  |  |  |
| NEL/BSW | 4 | 70,804,593 | 70,834,758 |  |  |  |  |
| GIR/BSW | 4 | 73,396,066 | 73,553,054 | ENSBTAG00000046430 | *ZNF804B* | Zinc finger protein 804B | 73,326,980-73,897,041 |
| GIR/ANG | 4 | 73,468,102 | 73,561,003 |  |  |  |  |
| NEL/BSW | 4 | 73,483,619 | 73,529,284 |  |  |  |  |
| NEL/ANG | 4 | 73,512,503 | 73,542,817 |  |  |  |  |
| NEL/ANG | 4 | 92,097,557 | 92,149,328 | ENSBTAG00000030030 | *bta-mir-592* | Bos taurus miR-592 stem-loop | 92,126,804-92,126,899 |
| BSW/ANG | 5 | 24,661,397 | 24,789,309 | None | *-* | - | - |
| GIR/BSW |  | 24,726,883 | 24,832,947 |  |  |  |  |
| GIR/BSW |  | 42,277,521 | 42,277,521 |  |  |  |  |
| NEL/ANG |  | 45,402,110 | 45,451,680 |  |  |  |  |
| GIR/NEL |  | 47,169,668 | 47,203,636 |  |  |  |  |
| NEL/BSW |  | 47,193,853 | 47,209,338 |  |  |  |  |
| GIR/NEL | 5 | 48,655,139 | 49,351,919 | ENSBTAG00000044017 | *MSRB3* | methionine sulfoxide reductase B3 | 48,563,806-48,743,354 |
|  |  |  |  | ENSBTAG00000039435 | *LEMD3* | LEM domain containing 3 (Inner nuclear membrane protein Man1) | 48,773,272-48,844,474 |
|  |  |  |  | ENSBTAG00000014758 | *BT.63013* | WNT inhibitory factor 1 precursor | 48,917,722-49,009,466 |
|  |  |  |  | ENSBTAG00000011352 | *TBC1D30* | TBC1 domain family, member 30 | 49,181,675-49,265,076 |
|  |  |  |  | ENSBTAG00000017465 | *BT.20235* | N-acetylglucosamine-6-sulfatase precursor | 49,283,646-49,333,344 |
|  |  |  |  | ENSBTAG00000015646 | *RASSF3* | Ras association (RalGDS/AF-6) domain family member 3 | 49,348,494-49,352,530 |
|  |  |  |  | ENSBTAG00000000237 | *BT.105896* | Possible orthologue: siah E3 ubiquitin protein ligase 1 | 49,247,178-49,248,008 |
|  |  |  |  | ENSBTAG00000045248 | *bta-mir-2429* | Bos taurus miR-2429 stem-loop | 48,952,298-48,952,372 |
|  |  |  |  | ENSBTAG00000042928 | *U6* | U6 spliceosomal RNA | 48,996,775-48,996,881 |
| GIR/NEL | 5 | 74,254,592 | 74,258,355 | ENSBTAG00000020125 | *RBFOX2* | RNA binding protein, fox-1  homolog 2 | 74,257,475-74,473,002 |
|  |  | 74,301,535 | 74,301,535 |  |  |  |  |
| GIR/ANG | 5 | 77,585,953 | 77,643,297 | ENSBTAG00000005453 | *FGD4* | FYVE, RhoGEF and PH domain containing 4 | 77,608,406-77,682,214 |
|  |  |  |  | ENSBTAG00000042195 | *U6* | U6 spliceosomal RNA | 77,638,003-77,638,109 |
| NEL/ANG | 5 | 77,631,251 | 77,833,386 | ENSBTAG00000005453 | *FGD4* | FYVE, RhoGEF and PH domain containing 4 | 77,608,406-77,682,214 |
|  |  |  |  | ENSBTAG00000042195 | *U6* | U6 spliceosomal RNA | 77,638,003-77,638,109 |
| BSW/ANG | 5 | 105,483,551 | 105,638,521 | ENSBTAG00000026522 | Novel Gene | Possible orthologue: ELOVL fatty acid elongase 1 | 105,562,079-105,568,210 |
|  |  |  |  | ENSBTAG00000007553 | *BT.53878* | Potassium voltage-gated channel subfamily A member 5 | 105,538,144-105,540,341 |
| BSW/ANG | 5 | 114,476,400 | 114,477,477 | None | *-* | - | - |
| GIR/ANG | 6 | 1,129,004 | 1,201,370 |  |  |  |  |
| NEL/ANG | 6 | 1,169,452 | 1,207,911 |  |  |  |  |
| GIR/NEL | 6 | 33,534,920 | 33,665,545 |  |  |  |  |
| BSW/ANG | 6 | 37,304,693 | 37,527,142 | ENSBTAG00000011187 | *FAM13A* | Protein FAM13A, family with sequence similarity 13, member A | 37,355,568-37,457,493 |
|  |  |  |  | ENSBTAG00000010120 | *HERC3* | Probable E3 ubiquitin-protein ligase HERC3 | 37,479,804-37,609,959 |
|  |  |  |  | ENSBTAG00000010128 | *NAP1L5* | Nucleosome assembly protein 1-like 5 | 37,509,213-37,511,139 |
| BSW/ANG | 6 | 37,642,525 | 37,811,938 | ENSBTAG00000020541 | *BT.67700* | Protein preY, mitochondrial precursor | 37,677,099-37,679,897 |
|  |  |  |  | ENSBTAG00000020538 | *HERC5* | E3 ISG15--protein ligase HERC5 | 37,683,713-37,728,536 |
|  |  |  |  | ENSBTAG00000020536 | *HERC6* | HECT and RLD domain containing E3 ubiquitin protein ligase family member 6 | 37,736,138-37,793,279 |
| GIR/BSW | 6 | 39,681,238 | 39,769,114 | None | *-* | - | - |
| BSW/ANG |  | 39,690,568 | 39,746,201 |  |  |  |  |
| BSW/ANG |  | 40,658,132 | 40,742,457 |  |  |  |  |
| GIR/NEL |  | 54,209,812 | 54,244,211 |  |  |  |  |
| GIR/BSW | 6 | 61,978,028 | 61,996,242 | ENSBTAG00000010677 | *LIMCH1* | LIM and calponin homology  domains 1 | 61,872,553-62,222,095 |
| GIR/BSW | 6 | 62,165,394 | 62,554,136 |  |  |  |  |
|  |  |  |  | ENSBTAG00000044166 | *BT.46280* | Uncharacterized protein | 62,295,688-62,298,730 |
|  |  |  |  | ENSBTAG00000043958 | *TMEM33* | Transmembrane protein 33 | 62,464,346-62,478,760 |
|  |  |  |  | ENSBTAG00000002550 | Novel gene | Known pseudogene | 62,506,125-62,506,524 |
|  |  |  |  | ENSBTAG00000020367 | *SLC30A9* | Solute carrier family 30 (zinc transporter), member 9 | 62,509,248-62,594,616 |
|  |  |  |  | ENSBTAG00000044640 | Novel gene | Known miRNA | 62,343,691-62,343,767 |
|  |  |  |  | ENSBTAG00000042558 | *7SK* | 7SK RNA | 62,225,327-62,225,649 |
| NEL/BSW | 6 | 62,185,083 | 62,476,880 | ENSBTAG00000010677 | *LIMCH1* | LIM and calponin homology domains 1 | 61,872,553-62,222,095 |
|  |  |  |  | ENSBTAG00000044166 | *BT.46280* | Uncharacterized protein | 62,295,688-62,298,730 |
|  |  |  |  | ENSBTAG00000043958 | *TMEM33* | Transmembrane protein 33 | 62,464,346-62,478,760 |
|  |  |  |  | ENSBTAG00000044640 | Novel gene | Known miRNA | 62,343,691-62,343,767 |
|  |  |  |  | ENSBTAG00000042558 | *7SK* | 7SK RNA | 62,225,327-62,225,649 |
| BSW/ANG | 6 | 62,304,587 | 62,334,908 | None | *-* | - | - |
| GIR/ANG | 6 | 64,544,058 | 64,559,290 |  |  |  |  |
| GIR/NEL | 6 | 66,750,850 | 66,787,589 |  |  |  |  |
| GIR/NEL | 6 | 77,355,924 | 77,429,402 |  |  |  |  |
| GIR/NEL | 6 | 81,372,213 | 81,431,979 |  |  |  |  |
| GIR/BSW | 6 | 81,574,642 | 81,625,162 | ENSBTAG00000024826 | *TECRL* | Trans-2,3-enoyl-CoA reductase-like | 81,511,554-81,653,990 |
| GIR/ANG |  | 81,580,533 | 81,637,706 |  |  |  |  |
| NEL/BSW |  | 81,582,379 | 81,649,283 |  |  |  |  |
| NEL/ANG |  | 81,585,226 | 81,673,521 |  |  |  |  |
| GIR/BSW | 6 | 93,624,872 | 93,729,681 | ENSBTAG00000021372 | *SEPT11* | Septin-11 | 93,528,980-93,628,589 |
|  |  |  |  | ENSBTAG00000000599 | *CCNI* | Cyclin-I | 93,633,306-93,672,214 |
|  |  |  |  | ENSBTAG00000037304 | Novel gene | Known miRNA | 93,691,971-93,692,054 |
| NEL/BSW | 6 | 93,658,078 | 93,658,078 | ENSBTAG00000000599 | *CCNI* | Cyclin-I | 93,633,306-93,672,214 |
| GIR/NEL | 7 | 45,509,478 | 45,509,478 | ENSBTAG00000002305 | *PCSK4* | Proprotein convertase subtilisin/kexin type 4 | 45,508,320-45,513,924 |
| BSW/ANG | 7 | 53,792,514 | 53,993,493 | ENSBTAG00000037885 | *PCDHB1* | Protocadherin beta 1 | 53,853,039-53,855,495 |
|  |  |  |  | ENSBTAG00000045858 | Novel gene | DnaJ (Hsp40) homolog, subfamily B, member 1 | 53,859,676-53,860,389 |
|  |  |  |  | ENSBTAG00000045603 | *PCDHB4* | Protocadherin beta 4 | 53,908,354-53,910,744 |
|  |  |  |  | ENSBTAG00000047478 | *PCDHB6* | Protocadherin beta 6 | 53,933,784-53,936,171 |
|  |  |  |  | ENSBTAG00000039433 | Uncharacterized protein | Protocadherin-psi1; | 53,946,908-53,949,301 |
|  |  |  |  | ENSBTAG00000046262 | *PCDHB7* | Protocadherin beta 7 | 53,973,806-53,976,193 |
|  |  |  |  | ENSBTAG00000045859 | *PCDHB13* | Protocadherin beta 13 | 53,979,051-53,981,402 |
|  |  |  |  | ENSBTAG00000015453 | *PCDHB16* | Protocadherin beta 16 | 53,986,019-53,988,346 |
|  |  |  |  | ENSBTAG00000045743 | *PCDHB10* | Protocadherin beta 10 | 53,990,709-53,993,078 |
| GIR/NEL | 7 | 65,840,794 | 65,909,733 | None | *-* | - | - |
| NEL/ANG | 7 | 98,496,280 | 98,499,038 | ENSBTAG00000000874 | *CAST* | Calpastatin isoform I - CAST gene | 98,444,979-98,581,253 |
| GIR/NEL | 8 | 43,687,157 | 43,800,528 | ENSBTAG00000008062 | *DMRT2* | Doublesex and mab-3 related transcription factor 2 | 43,780,986-43,787,918 |
| GIR/BSW | 8 | 46,292,757 | 46,328,656 | ENSBTAG00000033396 | *C8H9orf135* | Uncharacterized protein C9orf135 homolog | 46,235,396-46,339,984 |
| GIR/ANG |  | 46,309,661 | 46,327,604 |  |  |  |  |
| NEL/BSW |  | 46,312,788 | 46,316,213 |  |  |  |  |
| GIR/BSW | 9 | 936,172 | 992,177 | None | *-* | - | - |
| NEL/BSW | 9 | 965,514 | 984,151 |  |  |  |  |
| NEL/BSW | 9 | 7,180,050 | 7,301,202 |  |  |  |  |
| GIR/BSW | 9 | 7,262,920 | 7,279,953 |  |  |  |  |
| GIR/BSW | 9 | 11,088,402 | 11,106,194 |  |  |  |  |
| GIR/ANG | 9 | 11,092,240 | 11,106,194 |  |  |  |  |
| NEL/ANG | 9 | 49,905,617 | 49,915,316 | ENSBTAG00000020482 | *ASCC3* | activating signal cointegrator 1 complex subunit 3 | 49,714,504-50,103,135 |
| BSW/ANG | 9 | 62,351,190 | 62,355,569 | None | *-* | - | - |
| BSW/ANG | 9 | 94,961,885 | 95,018,357 | ENSBTAG00000002728 | *ARID1B* | AT rich interactive domain 1B | 94,882,344-95,271,883 |
|  |  |  |  | ENSBTAG00000037085 | *5S_rRNA* | 5S ribosomal RNA | 94,976,406-94,976,530 |
| NEL/BSW | 9 | 104,838,415 | 104,854,354 | None | *-* | - | - |
| NEL/BSW | 10 | 45,746,569 | 45,847,298 | ENSBTAG00000016823 | *CSNK1G1* | Casein kinase I isoform gamma-1 | 45,717,384-45,867,730 |
| GIR/NEL | 10 | 58,581,505 | 58,581,505 | ENSBTAG00000047868 | *BT.65683* | Tropomodulin 3 (ubiquitous) | 58,576,206-58,581,557 |
|  |  |  |  | ENSBTAG00000019601 | *TMOD3* | Novel protein coding | 58,569,062-58,646,799 |
| GIR/ANG | 11 | 21,557,961 | 21,687,024 | ENSBTAG00000024044 | *BT.38522* | Cyclin-dependent kinase-like 4 (CDKL4) | 21,526,906-21,574,655 |
|  |  |  |  | ENSBTAG00000016442 | *MAP4K3* | Mitogen-activated protein kinase kinase kinase kinase 3 | 21,579,432-21,761,348 |
|  |  |  |  | ENSBTAG00000043391 | *U6* | U6 spliceosomal RNA | 21,594,042-21,594,151 |
| GIR/NEL | 11 | 21,616,139 | 21,684,583 | ENSBTAG00000016442 | *MAP4K3* | Mitogen-activated protein kinase kinase kinase kinase 3 | 21,579,432-21,761,348 |
| NEL/BSW | 11 | 34,930,536 | 34,930,536 | None | *-* | - | - |
| BSW/ANG | 11 | 78,802,288 | 78,893,100 | ENSBTAG00000020894 | *LAPTM4A* | Lysosomal-associated transmembrane protein 4A | 78,862,495-78,880,461 |
|  |  |  |  | ENSBTAG00000020893 | *MATN3* | Matrilin 3 | 78,889,151-78,907,349 |
| GIR/BSW | 12 | 41,634,760 | 41,915,116 | ENSBTAG00000032979 | Novel gene | Uncharacterized protein | 41,907,041-41,907,707 |
| NEL/BSW | 12 | 41,664,252 | 41,803,562 | None | *-* | - | - |
| NEL/ANG | 12 | 41,731,150 | 41,761,556 |  |  |  |  |
| GIR/ANG | 12 | 41,788,070 | 41,814,404 |  |  |  |  |
| GIR/ANG | 12 | 41,987,029 | 42,007,452 |  |  |  |  |
| NEL/ANG | 12 | 60,197,449 | 60,278,968 |  |  |  |  |
| NEL/BSW | 12 | 60,221,415 | 60,250,304 |  |  |  |  |
| GIR/ANG | 12 | 60,222,348 | 60,277,487 |  |  |  |  |
| GIR/ANG | 12 | 62,275,841 | 62,281,272 |  |  |  |  |
| NEL/BSW | 13 | 23,987,726 | 24,071,436 | ENSBTAG00000018033 | *PIP4K2A* | Phosphatidylinositol-5-phosphate 4-kinase, type II, alpha | 23,900,873-24,085,889 |
| GIR/BSW | 13 | 24,025,913 | 24,054,124 |  |  |  |  |
| GIR/BSW | 13 | 40,779,594 | 40,837,102 | ENSBTAG00000042976 | *SNORA70* | Small nucleolar RNA SNORA70 | 40,793,316-40,793,450 |
| BSW/ANG | 14 | 11,831,317 | 11,877,078 | ENSBTAG00000020801 | *FAM49B* | Protein FAM49B | 11,839,259-11,871,079 |
| BSW/ANG | 14 | 26,811,770 | 26,826,652 | ENSBTAG00000004954 | *TOX* | Thymocyte selection-associated high mobility group box protein TOX | 26,631,190-26,941,726 |
| NEL/BSW | 14 | 27,413,044 | 27,451,053 | None | *-* | - | - |
| NEL/BSW | 15 | 663,684 | 677,309 |  |  |  |  |
| GIR/NEL | 15 | 31,280,178 | 31,405,516 | ENSBTAG00000021338 | *OAF* | OAF homolog (Drosophila) | 31,312,383-31,330,638 |
| BSW/ANG | 15 | 40,442,281 | 40,511,298 | ENSBTAG00000032657 | *TEAD1* | TEA domain family member 1 (SV40 transcriptional enhancer factor) | 40,303,805-40,482,346 |
| NEL/BSW | 15 | 51,762,198 | 51,925,201 | ENSBTAG00000013111 | *RRM1* | Ribonucleoside-diphosphate reductase M1 chain | 51,801,715-51,843,018 |
|  |  |  |  | ENSBTAG00000013109 | *BT.31041* | Stromal interaction molecule 1 precursor (STIM1) | 51,845,122-52,049,569 |
|  |  |  |  | ENSBTAG00000035686 | Novel gene | Homologous: olfactory receptor Olr40 | 51,778,327-51,779,310 |
|  |  |  |  | ENSBTAG00000005070 | Novel gene | Homologous: Olfr543 olfactory receptor 543 | 51,796,620-51,797,639 |
| NEL/ANG | 15 | 51,800,901 | 51,937,349 | ENSBTAG00000013111 | *RRM1* | Ribonucleoside-diphosphate reductase M1 chain | 51,801,715-51,843,018 |
|  |  |  |  | ENSBTAG00000013109 | *BT.31041* | Stromal interaction molecule 1 precursor (STIM1) | 51,845,122-52,049,569 |
| GIR/ANG | 15 | 51,846,443 | 51,906,377 |  |  |  |  |
| GIR/NEL | 15 | 52,400,296 | 52,507,718 | ENSBTAG00000014167 | *BT.21603* | RING finger protein 121 | 52,330,181-52,413,459 |
|  |  |  |  | ENSBTAG00000027676 | *IL18BP* | Interleukin 18 binding protein | 52,416,834-52,418,796 |
|  |  |  |  | ENSBTAG00000018449 | *NUMA1* | Nuclear mitotic apparatus protein 1 | 52,419,502-52,450,409 |
|  |  |  |  | ENSBTAG00000017964 | *LRRC51* | Leucine-rich repeat-containing protein 51 | 52,492,121-52,508,047 |
| GIR/NEL | 15 | 53,652,608 | 53,698,708 | ENSBTAG00000016494 | *RELT* | RELT tumor necrosis factor receptor | 53,663,176-53,669,426 |
|  |  |  |  | ENSBTAG00000016497 | *FAM168A* | Family with sequence similarity 168, member A | 53,678,628-53,697,584 |
| BSW/ANG | 15 | 56,260,041 | 56,314,375 | ENSBTAG00000016355 | *UVRAG* | UV radiation resistance associated gene | 55,992,137-56,317,353 |
| GIR/ANG | 15 | 71,508,857 | 71,539,892 | None | *-* | - | - |
| NEL/ANG | 15 | 71,511,957 | 71,519,687 |  |  |  |  |
| BSW/ANG | 15 | 72,764,357 | 72,779,774 |  |  |  |  |
| NEL/BSW | 16 | 37,305,713 | 37,321,820 |  |  |  |  |
| GIR/BSW | 16 | 37,321,820 | 37,321,820 |  |  |  |  |
| BSW/ANG | 16 | 38,288,836 | 38,288,836 | ENSBTAG00000014793 | *C1orf112* | Chromosome 1 open reading frame 112 | 38,250,081-38,299,757 |
| GIR/ANG | 16 | 42,747,137 | 42,839,388 | ENSBTAG00000020700 | *CLCN6* | Chloride channel, voltage-sensitive 6 | 42,719,367-42,750,151 |
|  |  |  |  | ENSBTAG00000020698 | *BT.26830* | Methylenetetrahydrofolate reductase | 42,750,520-42,765,210 |
|  |  |  |  | ENSBTAG00000009386 | *C1orf167* | Chromosome 1 open reading frame 167 | 42,765,851-42,788,588 |
|  |  |  |  | ENSBTAG00000009384 | *AGTRAP* | Type-1 angiotensin II receptor-associated protein | 42,806,430-42,825,905 |
| NEL/ANG | 16 | 58,296,357 | 58,374,991 | None | *-* | - | - |
| GIR/ANG | 16 | 58,308,395 | 58,353,455 |  |  |  |  |
| GIR/BSW | 17 | 22,343,829 | 22,364,546 |  |  |  |  |
| GIR/BSW | 17 | 40,221,735 | 40,252,780 |  |  |  |  |
| NEL/BSW | 17 | 49,057,875 | 49,083,822 | ENSBTAG00000002950 | *TMEM132D* | Transmembrane protein 132D | 49,075,827-49,090,245 |
| NEL/ANG | 17 | 58,279,835 | 58,299,725 | None | *-* | - | - |
| GIR/NEL | 17 | 58,810,919 | 58,841,608 |  |  |  |  |
| GIR/BSW | 18 | 12,360,341 | 12,360,341 |  |  |  |  |
| GIR/BSW | 18 | 12,671,466 | 12,738,699 |  |  |  |  |
| BSW/ANG | 18 | 15,701,264 | 15,747,337 | ENSBTAG00000004805 | *ITFG1* | T-cell immunomodulatory protein precursor | 15,625,555-15,928,962 |
| BSW/ANG | 18 | 42,393,422 | 42,411,525 | None | *-* | - | - |
| NEL/ANG | 18 | 46,966,636 | 46,980,139 | ENSBTAG00000015004 | *TBCB* | Tubulin-folding cofactor B | 46,963,844-46,972,220 |
| GIR/NEL | 20 | 21,961,802 | 22,047,292 | ENSBTAG00000012124 | *GPBP1* | Vasculin | 22,033,857-22,098,038 |
| GIR/NEL | 20 | 40,153,984 | 40,330,326 | ENSBTAG00000012558 | *ADAMTS12* | ADAM metallopeptidase with thrombospondin type 1 motif 12 precursor | 39,913,085-40,304,028 |
| GIR/ANG | 20 | 40,256,197 | 40,259,027 |  |  |  |  |
| NEL/BSW | 21 | 16,283,658 | 16,302,869 | ENSBTAG00000027074 | *SV2B* | synaptic vesicle glycoprotein 2B | 16,214,670-16,460,490 |
| GIR/NEL | 21 | 34,625,629 | 34,647,179 | ENSBTAG00000011446 | *SEMA7A* | semaphorin 7A precursor, GPI membrane anchor | 34,626,181-34,651,240 |
| NEL/BSW | 21 | 44,627,082 | 44,667,640 | None | *-* | - | - |
| GIR/BSW | 21 | 44,633,485 | 44,679,996 | ENSBTAG00000004462 | *NPAS3* | neuronal PAS domain protein 3 | 44,674,273-44,703,824 |
| GIR/NEL | 21 | 60,490,921 | 60,601,371 | None | *-* | - | - |
| BSW/ANG | 22 | 33,849,503 | 34,007,257 | ENSBTAG00000009541 | *SUCLG2* | Succinyl-CoA ligase [GDP-forming] subunit beta, mitochondrial | 33,977,312-34,251,728 |
| BSW/ANG | 23 | 375,470 | 629,385 | ENSBTAG00000043990 | *KHDRBS2* | KH domain containing, RNA binding, signal transduction associated 2 | 184,673-864,355 |
| BSW/ANG | 23 | 756,762 | 853,782 |  |  |  |  |
| BSW/ANG | 24 | 37,711,307 | 37,890,877 | ENSBTAG00000019585 | *MYOM1* | myomesin 1, 185kDa | 37,673,546-37,791,756 |
|  |  |  |  | ENSBTAG00000016024 | *MYL9* | Myosin regulatory light polypeptide 9 | 37,820,258-37,829,300 |
|  |  |  |  | ENSBTAG00000026266 | *MYL12B* | Myosin regulatory light chain 12B | 37,834,593-37,910,130 |
| GIR/NEL | 24 | 45,334,592 | 45,407,249 | None | *-* | - | - |
| BSW/ANG | 25 | 16,327,370 | 16,334,028 |  |  |  |  |
| BSW/ANG | 25 | 16,368,012 | 16,368,012 |  |  |  |  |
| BSW/ANG | 25 | 16,388,431 | 16,402,215 |  |  |  |  |
| NEL/ANG | 26 | 25,608,100 | 25,625,024 |  |  |  |  |
| GIR/ANG | 26 | 25,612,816 | 25,615,778 |  |  |  |  |
| BSW/ANG | 26 | 25,619,204 | 25,632,810 |  |  |  |  |
| NEL/BSW | 26 | 46,967,798 | 46,967,798 | ENSBTAG00000031890 | *DOCK1* | dedicator of cytokinesis 1 | 46,791,127-47,295,654 |
|  |  |  |  | ENSBTAG00000021015 | *FAM196A* | family with sequence similarity 196, member A | 46,963,427-47,002,009 |
| GIR/NEL | 26 | 50,987,258 | 51,054,320 | ENSBTAG00000019919 | *TTC40* | tetratricopeptide repeat domain 40 | 51,008,298-51,070,531 |
| NEL/BSW | 27 | 3,348,155 | 3,393,247 | None | *-* | - | - |
| GIR/BSW |  | 3,347,923 | 3,407,280 |  |  |  |  |
| BSW/ANG | 27 | 34,670,524 | 34,721,974 | ENSBTAG00000020602 | *BT.19792* | indoleamine 2,3-dioxygenase 1 (IDO1) | 34,686,577-34,699,480 |
|  |  |  |  | ENSBTAG00000005725 | *IDO2* | indoleamine 2,3-dioxygenase 2 | 34,707,901-34,774,452 |
| NEL/ANG | 29 | 25,106,243 | 25,161,229 | ENSBTAG00000018431 | *NAV2* | neuron navigator 2 | 25,049,904-25,356,586 |
| NEL/BSW | 29 | 40,196,311 | 40,240,704 | ENSBTAG00000013214 | *BT.88769* | Uncharacterized protein | 40,222,386-40,231,181 |
|  |  |  |  | ENSBTAG00000031483 | Novel gene | Known processed pseudogene | 40,214,778-40,215,227 |

* gene ID source and Position: ENSEMBL : http://www.ensembl.org/ ** Gene functions: NCBI Entrez Gene: http://www.ncbi.nlm.nih.gov/gene/
