# Supplementary material for: Assessing signatures of selection through variation in linkage disequilibrium between taurine and indicine cattle
Source: Genet Sel Evol. 2014 Mar 4;46(1):19. doi: 10.1186/1297-9686-46-19 (PMC4014805; doi:10.1186/1297-9686-46-19)
Supplement: Additional file 2 — Common signals with other Signatures of Selection studies. Common signals found between our analysis and previous signatures of selection regions reported in the literature; the breed comparison where the signal came from, the chromosome number, the base pair spanning position, the position of the common region, the reference to the authors and the gene name are given in the Table; several VarLD signals that coincide with the same signal from other studies are highlighted in yellow, while several signals from other authors that concur with one VarLD Signal are highlighted in blue [5,7,9,41,54,63,64,69-73]. [file 1297-9686-46-19-S2.doc]

| **Comparison** | **Chr** | **varLD signal position (bp)** | **Other signal position (bp)** | **Method** | **Author** | **Genes*** |
| --- | --- | --- | --- | --- | --- | --- |
| BSW/ANG | 1 | 12,175,001:12,258,890 | 3,654,000:12,740,000 | Smoothed Fst | Gautier et al. 2009 [70] | Novel miRNA |
|  |  |  | 12,000,000:13,000,000 | Fst (ϴs) | Qanbari et al. 2011 [54] |  |
| GIR/BSW | 1 | 70,765,503:70,792,506 | 70,500,000 | Fst (ϴs) | Qanbari et al. 2011 [54] | *leishmanolysin-like (metallopeptidase M8 family)* |
| GIR/ANG  NEL/BSW  GIR/BSW | 2 | 65,465,244:65,479,573  65,540,772:65,604,699  65,580,520:65,599,835 | 64,040,000:67,280,000 | Smoothed Fst | Gautier et al. 2009 [70] | *-* |
| GIR/ANG | 2 | 71,533,731:71,553,782 | 71,480,000:78,320,000 | Smoothed Fst | Gautier et al. 2009 [70] | *UPF0538 protein C2orf76 homolog* |
|  |  |  | 71,564,217 | Allele Frequency | Hayes et al. 2009 [5] |  |
|  |  |  | 70,000,000:73,000,000 | \|iHS\| | Qanbari et al. 2011 [54] |  |
| GIR/BSW | 3 | 14,993,410:15,069,437 | 12,390,000:19,720,000 | Smoothed Fst | Gautier et al. 2009 [70] | *GON-4-like protein,*  *MSTO1,*  *DAP3,*  *probable histone-lysine N-methyltransferase ASH1L,*  *Farnesyl pyrophosphate synthase,*  *PKLR,*  *EFNA1* |
| NEL/ BSW |  | 15,016,153:15,137,198 |  |  |  |  |
| GIR/ANG |  | 15,016,365:15,067,190 |  |  |  |  |
| NEL/ANG |  | 15,072,372:15,143,801 |  |  |  |  |
| NEL/ BSW |  | 15,271,777:15,311,541 |  |  |  |  |
| NEL/ANG |  | 15,299,719:15,400,121 |  |  |  |  |
| GIR/ANG |  | 15,342,320:15,400,959 |  |  |  |  |
| NEL/ANG |  | 15,523,608:15,552,545 |  |  |  |  |
| GIR/ANG | 4 | 70,487,854:70,570,667 | 66,290,000:75,270,000 | Smoothed Fst | Gautier et al. 2009 [70] | *zinc finger protein 804B,*  *U6* |
| GIR/ANG |  | 70,768,097:70,933,946 |  |  |  |  |
| NEL/ANG |  | 70,786,800:70,897,862 |  |  |  |  |
| NEL/ BSW |  | 70,804,593:70,834,758 |  |  |  |  |
| GIR/BSW |  | 73,396,066:73,553,054 |  |  |  |  |
| GIR/ANG |  | 73,468,102:73,561,003 |  |  |  |  |
| NEL/ BSW |  | 73,483,619:73,529,284 |  |  |  |  |
| NEL/ANG |  | 73,512,503:73,542,817 |  |  |  |  |
| GIR/BSW | 4  4  4  4 | 73,396,066:73,553,054 | 73,518,942; 73,527,945 | Rsb | Utsunomiya et al. 2013 [64] (Unpublished data) | *zinc finger protein 804B* |
| GIR/ANG |  | 73,468,102:73,561,003 |  |  |  |  |
| NEL/ BSW |  | 73,483,619:73,529,284 |  |  |  |  |
| NEL/ANG |  | 73,512,503:73,542,817 |  |  |  |  |
| GIR/NEL | 5 | 48,655,139:49,351,919 | 48,679,627:48,903,409 | MAF | Ramey et al. 2013 [41] | *MSRB3* |
|  |  |  | 47,890,000:54,390,000 | Smoothed Fst | Gautier et al. 2009 [70] | *MSRB3, LEMD3, wnt inhibitory factor 1 precursor, TBC1D30, GNS, RASSF3, possible orthologue: siah E3 ubiquitin protein ligase, Bos taurus miR-2429 stem-loop, U6* |
| NEL/ANG | 5 | 77,631,251:77,833,386 | 77,546,764:77,805,841 | EHH | Pan et al. 2013 [71] | *FGD4, U6* |
| BSW/ANG | 5 | 105,480,000:105,640,000 | 104,500,000:105,600,000 | iHs | The Bovine HapMap Consortium 2009 [9] | *ELOVL fatty acid elongase 1* |
| BSW/ANG | 5 | 114,476,400:114,477,477 | 110,800,000:118,700,000 | Compound diplotypes | Chan et al. 2010 [7] | *-* |
| GIR/NEL | 6 | 33,534,920:33,665,545 | 33,400,000:34,400,000 | iHs | The Bovine HapMap Consortium 2009 [9] | *-* |
| BSW/ANG | 6 | 37,304,693:37,527,142 | 34,000,000:37,600,000 | Compound diplotypes | Chan et al. 2010 [7] | *FAM13A,*  *HERC3,*  *NAP1L5* |
|  |  |  | 36,800,000:37,800,000 | iHs | The Bovine HapMap Consortium 2009 [9] |  |
|  |  |  | 37,433,000:38,756,000 | Smooth SNP-specific Fst | Flori et al. 2009 [63] |  |
| BSW/ANG | 6 | 37,642,525:37,811,938 | 36,800,000:37,800,000 | iHs | The Bovine HapMap Consortium 2009 [9] | *protein preY-mitochondrial precursor, HERC5. HERC6* |
| GIR/BSW | 6 | 61,978,028:61,996,242 | 61,750,000:62,750,000 | |iHS| | Qanbari et al. 2011 [54] | *LIM and calponin homology domains 1* |
| GIR/BSW | 6 | 62,165,394:62,554,136 | 62,000,000 | |iHS| | Qanbari et al. 2011 [54] | *LIM and calponin homology domains 1* |
|  |  |  | 62,200,296;62,203,090 | Rsb | Utsunomiya et al. 2013 [64] |  |
|  |  |  | 62,500,000 | \|iHS\| | Qanbari et al. 2011 [54] | *Novel Pseudogene, SLC30A9* |
| NEL/BSW | 6 | 62,185,083:62,476,880 | 62,200,296;62,203,090 | Rsb | Utsunomiya et al. 2013 [64] | *LIM and calponin homology domains 1* |
|  |  |  | 62,000,000 | \|iHS\| | Qanbari et al. 2011 [54] |  |
| GIR/ANG | 6 | 64,544,058:64,559,290 | 63,097,961:65,672,360 | CLL (Composite Log Likelihood) | Stella et al. 2010 [72] | *-* |
| GIR/NEL | 6 | 66,750,850:66,787,589 | 66,599,000:66,935,000 | smooth SNP-specific FST | Flori et al. 2009 [63] | *-* |
| GIR/NEL | 6 | 81,372,213:81,431,979 | 80,000,000:83,000,000 | |iHS| | Qanbari et al. 2011 [54] | *TECRL* |
| GIR/BSW |  | 81,574,642:81,625,162 |  |  |  |  |
| GIR/ANG |  | 81,580,533:81,637,706 |  |  |  |  |
| NEL/BSW |  | 81,582,379:81,649,283 |  |  |  |  |
| NEL/ANG |  | 81,585,226:81,673,521 |  |  |  |  |
| NEL/BSW | 6 | 93,658,078:93,658,078 | 89,360,000:105,400,000 | Smoothed Fst | Gautier et al. 2009 [70] | *Cyclin-I*  *Septin-11,*  *novel gene* |
| GIR/BSW |  | 93,624,872:93,729,681 |  |  |  |  |
| GIR/NEL | 7 | 45,509,478:45,509,478 | 45,439,468:45,828,427 | MAF | Ramey et al. 2013 [41] | *PCSK4* |
|  |  |  | 45,400,000:47,100,000 | iHs | The Bovine HapMap Consortium 2009 [9] |  |
| GIR/NEL | 7 | 65,840,794:65,909,733 | 65,230,000:67,120,000 | Smoothed Fst | Gautier et al. 2009 [70] |  |
| GIR/NEL | 8 | 43,687,157:43,800,528 | 41,460,000:49,560,000 | Smoothed Fst | Gautier et al. 2009 [70] | *DMRT2, C8H9orf135* |
| GIR/BSW |  | 46,292,757:46,328,656 |  |  |  |  |
| GIR/ANG |  | 46,309,661:46,327,604 |  |  |  |  |
| NEL/BSW |  | 46,312,788:46,316,213 |  |  |  |  |
| GIR/NEL | 8 | 43,687,157:43,800,528 | 40,500,000:47,300,000 | Compound diplotypes | Chan et al. 2010 [7] | *DMRT2, C8H9orf135* |
| GIR/BSW |  | 46,292,757:46,328,656 |  |  |  |  |
| GIR/ANG |  | 46,309,661:46,327,604 |  |  |  |  |
| NEL/BSW |  | 46,312,788:46,316,213 |  |  |  |  |
| BSW/ANG | 11 | 78,802,288:78,893,100 | 78,803,108 | Fst | Barendse et al. 2009 [69] |  |
| NEL/ANG | 12 | 60,197,449:60,278,968 | 60.000.000 | Fst (ϴs) | Qanbari et al. 2011 [54] | *-* |
| NEL/BSW |  | 60,221,415:60,250,304 |  |  |  |  |
| GIR/ANG |  | 60,222,348:60,277,487 |  |  |  |  |
| NEL/BSW | 13 | 23,987,726:24,071,436 | 23,600,000:24,700,000 | Rsb AFT⁄CGU | Gautier and Naves 2011 [73] | *PIP4K2A* |
| BSW/ANG | 14 | 11,831,317:11,877,078 | 9,072,000:13,530,000 | Smoothed Fst | Gautier et al. 2009 [70] | *FAM49B* |
| BSW/ANG | 14 | 26,811,770:26,826,652 | 23,900,000:30,100,000 | iHs | The Bovine HapMap Consortium 2009 [9] | *TOX* |
| NEL/BSW | 16 | 37,305,713:37,321,820 | 35,200,000:45,200,000 | Compound diplotypes | Chan et al. 2010 [7] |  |
| GIR/BSW |  | 37,321,820 |  |  |  |  |
| BSW/ANG |  | 38,288,836 |  |  |  | *C1ORF112* |
| GIR/ANG | 16 | 42,747,137:42,839,388 |  |  |  | *CLCN6,*  *Methylenetetrahydrofolate reductase, C1ORF167*  *CLCN6,*  *Methylenetetrahydrofolate reductase, C1ORF167* |
| GIR/ANG |  |  | 42,555,966:42,788,613 | MAF | Ramey et al. 2013 [41] |  |
| GIR/BSW | 17 | 40,221,735:40,252,780 | 39,500,000:40,500,000 | Fst (ϴs) | Qanbari et al. 2011 [54] |  |
| GIR/BSW | 18 | 12,360,341:12,360,341 | 12,300,000:13,300,000 | Rsb EUT/CGU - AFT ⁄CGU | Gautier and Naves 2011 [73] | *-* |
| GIR/BSW | 18 | 12,671,466:12,738,699 | 12,300,000:13,300,000 | Rsb EUT/CGU - AFT ⁄CGU | Gautier and Naves 2011 [73] | *-* |
| GIR/BSW | 18 | 12,671,466:12,738,699 | 12,500,000 | |iHS| | Qanbari et al. 2011 [54] | *-* |
| BSW/ANG | 18 | 15,701,264:15,747,337 | 14,900,000:21,100,000 | EHH | Chan et al. 2010 [7] | *ITFG1* |
| BSW/ANG | 18 | 42,393,422:42,411,525 | 42,000,000 | Fst (ϴs) | Qanbari et al. 2011 [54] | *-* |
| GIR/NEL | 21 | 34,625,629:34,647,179 | 33,852,145:35,259,414 | CLL (Composite Log Likelihood) | Stella et al. 2010 [72] | *SEMA7A* |
| BSW/ANG | 22 | 33,849,503:34,007,257 | 32,320,000:38,560,000 | Smoothed Fst | Gautier et al. 2009 [70] | *SUCLG2* |
| GIR/NEL | 24 | 45,334,592:45,407,249 | 44,978,575:45,664,478 | CLL (Composite Log Likelihood) | Stella et al. 2010 [72] | *-* |

* gene ID source: ENSEMBL : http://www.ensembl.org/
